# Quantitative Design of Regulatory Elements Based on High-Precision Strength Prediction Using Artificial Neural Network

**DOI:** 10.1371/journal.pone.0060288

**Published:** 2013-04-01

**Authors:** Hailin Meng, Jianfeng Wang, Zhiqiang Xiong, Feng Xu, Guoping Zhao, Yong Wang

**Affiliations:** 1 Key Laboratory of Synthetic Biology, Institute of Plant Physiology and Ecology, Shanghai Institutes for Biological Sciences, Chinese Academy of Sciences, Shanghai, China; 2 State Key Laboratory of Bioreactor Engineering, East China University of Science and Technology, Shanghai, China; Niels Bohr Institute, Denmark

## Abstract

Accurate and controllable regulatory elements such as promoters and ribosome binding sites (RBSs) are indispensable tools to quantitatively regulate gene expression for rational pathway engineering. Therefore, *de novo* designing regulatory elements is brought back to the forefront of synthetic biology research. Here we developed a quantitative design method for regulatory elements based on strength prediction using artificial neural network (ANN). One hundred mutated Trc promoter & RBS sequences, which were finely characterized with a strength distribution from 0 to 3.559 (relative to the strength of the original sequence which was defined as 1), were used for model training and test. A precise strength prediction model, NET90_19_576, was finally constructed with high regression correlation coefficients of 0.98 for both model training and test. Sixteen artificial elements were *in silico* designed using this model. All of them were proved to have good consistency between the measured strength and our desired strength. The functional reliability of the designed elements was validated in two different genetic contexts. The designed parts were successfully utilized to improve the expression of BmK1 peptide toxin and fine-tune deoxy-xylulose phosphate pathway in *Escherichia coli*. Our results demonstrate that the methodology based on ANN model can *de novo* and quantitatively design regulatory elements with desired strengths, which are of great importance for synthetic biology applications.

## Introduction

The coming era of synthetic biology aims at design and construction of complex biological networks to achieve our special goals (e.g., high-level production of clinically valuable natural products), which requires fine-tuning gene expression in the cellular networks to achieve an expected metabolic behaviour [1], [2]. Genetic elements with desired strengths/activities, e.g., promoters/RBSs for transcriptional/translational controls, are the most important tools to accurately control the expression of rate-limiting genes in an engineered system. In the last decade, an array of randomly mutated or synthetic promoter libraries with a wide range of strength have been constructed and applied to control protein expression or pathway engineering in *E. coli* and yeast [3], [4], [5]. However, acquisition of a controllable regulatory element from a random library needs laborious screening and multifaceted characterizations to ensure homogeneity at the single-cell level [5], especially in the situation of multiple genes regulated at different expression levels in one system. More recently, great advances in synthetic biology and its applications in engineering pathways and producing valuable chemicals in microbes have brought back the sequence-activity modelling approaches to the forefront. Researchers have put immense interests to decipher the ‘regulatory code’ (e.g., −10/−35 region, RBS region) that translates DNA sequences into expression strength [6], [7], [8], [9], and built quantitative models for strength prediction and rational design of regulatory elements [10], [11], [12], [13]. For instance, based on position weight matrix (PWM) models, Rhodius VA *et al* [12] scored various motifs of *E. coli σ* ^E^ binding promoters and correlated promoter scores with *in vitro* and *in vivo* measured strength, the correlation coefficient values (*R*) ranged from 0.57 to 0.77 for *in vitro* and *in vivo* strength fit was achieved. Besides promoter, Salis HM *et al* [10] targeted translation initiation process and developed a equilibrium statistical thermodynamics model for designing synthetic RBSs (linear regression *R* ^2^ ranged from 0.54 to 0.91), which correlates the Gibbs free energy variation of translation initiation with the translation rate. The above methods mainly targeting feature motifs or key processes have reached a certain point of success. But as Jensen *et al* [14] and De Mey M *et al* [11] indicated, promoter strength could not be simply linked to anomalies in the feature motifs such as −10 box and/or −35 box, and to the length of spacer. Therefore, De Mey M *et al* [11] established a correlation between the entire sequence and strength by applying partial least squares (PLS) regression method. This model exhibits promising applications for quantitative strength prediction and rational design of promoters, but still has great potential to improve its accuracy. Hence, building precise computational models that can predict the activity of regulatory elements and quantitatively design elements with desired strength is still a real challenge in gene expression area over decades.

Aforementioned quantitative prediction models commonly use linear regression analysis or its derivative methods (e.g., linear correlation of data after logarithm processing) to simplify the complex process for model construction. Thus, it is hard to well reflect the complex non-linear relationship between the sequences and their strengths, which results in a low prediction accuracy and poor generality. In addition, these models are supposed to have the potential, but have not been further developed into *in silico* methods for *de novo* design of elements with desired strength. In contrast to the above methods, we introduced a non-linear modelling methodology, artificial neural network (ANN), to address these issues. ANN is essentially a mathematical model constructed by simulation of the structure and function of human brain neural networks [15], [16]. It can be adapted to continuously change the network structure based on input/output information during learning phase, which could reflect the non-linear relationships between quantitative characteristics and related qualitative performance in complex phenomena. Thus, ANNs have been widely used to various biological research fields such as protein structure and stability prediction [17], [18], [19], RNA secondary structure prediction [20], as well as promoter recognition and structure analysis [21], [22], [23], [24], [25], [26], [27], [28]. In this work, we constructed a high-performance ANN model to directly predict the strength of regulatory element from its sequence. Based on this model, we further developed an effective computational platform for quantitative design of novel regulatory elements with desired properties for synthetic biology applications.

## Materials and Methods

### Strains, plasmids, reagents and general manipulation

All strains and plasmids involved in this study are listed in Table 1. *E. coli* DH10B was used for library construction and strength quantification. *E. coli* BL21(DE3) was served for BmK1 expression and amorphadiene biosynthesis. Enzymes & reagents for DNA manipulation and bacteria culture were purchased from New England Biolabs, Takara, or Oxoid. All primers, designed sequences, codon optimized [29] peptide BmK1 (*bmk*1, Genbank: AAD39510), and amorphadiene synthase (*ads*, Genbank: AAF98444) genes [30], were synthesized by Generay Biotech Ltd. (Shanghai, China). Antibiotics were added according to the resistance marker of plasmids in each culture. The working concentration of ampicillin and kanamycin were 100 mg/L and 50 mg/L, respectively.

**Table 1:** Strains and plasmids in this study.

Strains & plasmids	Relevant characteristics	Source
DH10B	F- *mcr*A Δ(*mrr*-*hsd*RMS-*mcr*BC) φ80*lac*ZΔM15 Δ*lac*X74 *rec*A1 *end*A1 *ara*D139 Δ(*ara*, *leu*)7697 *gal*U *gal*K *λ- rps*L *nup*G	Invitrogen
BL21(DE3)	F*- omp*T *hsd*S (*r*BB*-m*B*-*) *gal dcm* (DE3)	EMD4 Biosciences
pTrcHis2B	Ampicillin resistance marker, Trc promoter	Invitrogen
pJF07	Plasmid pTrcHis2B carrying a *gfp* gene at BamHI/EcoRI sites	This study
pET28a-*isp*A	pET28a derived plasmid carrying a *isp*A gene at NcoI/EcoRI sites	This study
pET21c-*ads*	pET21c derived plasmid carrying a *ads* gene at NdeI/EcoRI sites	This study
pET28a-*isp*A-*ads*	pET28a derived plasmid carrying *isp*A, *ads* for amorphadiene production	This study
s14/s05/s21-*gfp*	Three pTrcHis2B derived plasmids carrying synthetic promoters s14 (0.56), s05 (1.00) and s21 (2.50) followed by a *gfp* gene at BamHI/EcoRI sites, respectively	This study
s14/s05/s21-*bmk*1	Three pTrcHis2B derived plasmids carrying synthetic promoters s14 (0.56), s05 (1.00) and s21 (2.50) followed by a *bmk*1 gene at NcoI/HindIII sites, respectively	This study
s14/s05/s21-*dxs*	Three pTrcHis2B derived plasmids carrying synthetic promoters s14 (0.56), s05 (1.00) and s21 (2.50) followed by a *dxs* gene at NcoI/EcoRI sites, respectively	This study
pTrcHis2B-*dxs*	pTrcHis2B derived plasmid carrying a *dxs* gene at NcoI/EcoRI sites	This study

Reporter plasmid pJF07 was created by inserting a *gfp* gene [5] into the *Bam*HI & *Eco*RI sites of pTrcHis2B. To create plasmids s14/s05/s21-*bmk*1, the *bmk*1 gene was inserted into plasmids s14/s05/s21-*gfp* to replace the *gfp* gene. The *dxs* gene was obtained by PCR using primers *dxs*F (5′-CATGCCATGGGCATGAGTTTTGATATTGCCAAATACCCG-3′) and *dxs*R (5′-CCGGAATTCACTAGTTTATGCCAGCCACCTT-3′) and genomic DNA of *E. coli* K12 MG1655 as template. The isolated *dxs* gene fragment was cloned into the *Nco*I & *Eco*RI sites of pTrcHis2B to create plasmid pTrcHis2B-*dxs*, or inserted into s14/s05/s21-*gfp* to replace the *gfp* for creation of plasmids s14/s05/s21-*dxs* respectively. The *ads* gene was inserted into the *Nde*I & *Eco*RI sites of pET21c to create plasmid pET21c-*ads*. The *ispA* gene was isolated by PCR using primers (5′-CATGCCATGGGCATGGACTTTCCGCAGCAACTCGAAG-3′) and (5′-CCGGAATTCACTAGTTTATTTATTACGCTGGATGATGTAG-3′) and the genomic DNA of *E. coli* K12 MG1655 as template. The product of *ispA* was inserted into the *Nco*I & *Eco*RI sites of pET28a to create pET28a-*ispA*. The *Xba*I & *Eco*RI excised fragment of pET21c-*ads* was inserted into the *Spe*I & *Eco*RI sites of pET28a-*ispA* to create pET28a*-ispA-ads*. All standard DNA manipulations were performed as described by Sambrook *et al.* [31].

### Library construction and characterization

Random mutagenesis of the wild-type Trc promoter & RBS sequence was performed by error-prone PCR using primers TrcF (5′-ATAAGAATGCGGCCGCAACGGTTCTGGCAAATATTCTGAAAT-3′, the restriction site is underlined. The same below) and TrcR (5′-TCCTTTACGCATTGGATCCATGG-3′) and plasmid pJF07 as template according to the Kit's instruction (JBS Error-Prone Kit PP101, Jena Bioscience). The reporter plasmid skeleton was PCR amplified by PrimeSTAR DNA polymerase using primers pJF07F (5′-CGGGATCCAATGCGTAAAGGAGAAGAAC-3′) and pJF07R (5′-ATAAGAATGCGGCCGCATGATGTCGGCGCAAAAAACATTATC-3′) and plasmid pJF07 as template. PCR products of the reporter plasmid skeleton and Trc promoter & RBS excised by *Not*I & *Bam*HI were ligated and transformed into DH10B competent cells. The transformed culture were spread onto LB agar plate and cultivated overnight at 37°C for 16 hours.

For primary screening, transformants were picked out into the 48-deep-well plate and screened through gene fluorescent protein assay (excitation/emission wavelength = 485 nm/535 nm). The conditions for 48-deep-well plate cultivation were as follows: 0.5 ml LB medium with 0.1 mM IPTG in each 5 ml-well at 37°C and 250 rpm for 8 h during exponential phase. The OD_600 nm_ and green fluorescent signal of 100 µl culture was quantified in a 96-well plate reader (Multiskan FC Microplate Photometer, Thermo Scientific). For the convenience of comparison, we used the relative strength [30] to represent the strength of a mutated sequence, which was defined and calculated as
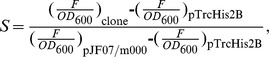
where 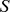 is the relative strength of the sequence, 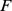 the fluorescent value; 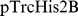 the blank control, and 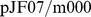 the wild-type Trc promoter & RBS.

One hundred clones with distributed strength were selected and cultivated overnight in LB broth and preserved in 20% glycerol at −80°C for seed culture. Fine quantification of the selected elements was performed in tube (15 mm×150 mm) and assayed by flow cytometry (FACSCalibur flow cytometer, Bection Dickinson). Seventy-five microliters of seed culture was innoculated into 1.5 ml LB with 0.1 mM IPTG and incubated at 37°C and 250 rpm for 3 h at exponential phase. The culture was cooled with ice bath and assayed using clone containing pTrcHis2B as blank control. Each clone was sampled with 20,000 events and the geometric mean (Gmean) of fluorescent signal was calculated using statistics. The relative strength value [32] compared with wild-type Trc promoter & RBS was calculated as
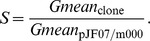



### Computational platform construction

Matlab 2012a (Mathworks Inc., http://www.mathworks.com/) ran on a personal computer with Microsoft Windows 7 64-bit (Microsoft Inc., http://www.microsoft.com/) operation system. Neural Network Toolbox within Matlab served as the basic tool for artificial neural network (ANN) model construction, data fitting and prediction. All programs used in this work were designed and run upon Neural Network Toolbox and Matlab environment.

### Cultivation of recombinant strains and products analysis

Peptide expression was performed in *E. coli* BL21(DE3) at 37°C and 250 rpm, induced with 0.1 mM IPTG at 0.6 of OD_600_ for 3 h, and analyszed by SDS-PAGE. The stained PAGE was imaged by Tanon 2500R gel-imaging system (Tanon Science & Technology Ltd., Shanghai, China). The relative content of BmK1 to total cellular protein was calculated by the GIS 1-D software (Tanon) according to the ratio of the intensity of target peptide band to that of all protein bands.

Recombinant strains *E. coli* BL21(DE3) harbouring plasmid pET28a-*ispA*-*ads* and s14-*dxs*, or s05-*dxs*, or s21-*dxs* were used for amorphadiene production. Shake-flask fermentation was performed using the following conditions: 2% of inoculation and 10 ml TB medium (12 g/L tryptone, 24 g/L yeast extract, 2.31 g/L KH_2_PO_4_, 12.54 g/L K_2_HPO_4_) in 100 ml shake-flask with 2% glycerol, 20% dodecane, and 0.1 mM IPTG at 28°C and 250 rpm for 3 days. After cultivation, dodecane phase was diluted using ethyl acetate to an appropriate concentration and analysed by GC-MS using caryophyllene (Sigma-Aldrich) as internal standard [30].

## Results

### Construction of Trc promoter & RBS strength library

**Figure 1:**
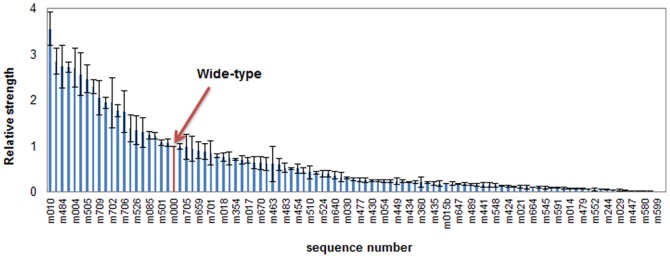
Relative strengths of the constructed Trc promoter & RBS library. The region of Trc promoter & RBS in pTrcHis2B is selected for random mutagenesis by error prone PCR, and mutants with various strength are obtained by detecting the fluorescent intensity of GFP after screening by 48-deep-well plates and flow cytometry assay.

Trc promoter is commonly used for protein expression in *E. coli* or other prokaryotic systems. To build and train the ANN models, we initially constructed and characterized a mutated Trc promoter library. Considering that protein expression is influenced by both transcription and translation processes, herein the DNA region of Trc promoter plus its RBS (224 bp in total) from a commercial plasmid pTrcHis2B was subjected to random mutagenesis. The mutagenesis rate of the library reached up to about 20%. After initial screening 4,000 clones, 100 mutants with uniformly distributed strengths were chosen to construct a strength-gradient library (Figure 1 and Text S1). All of the mutants were finely quantified and sequenced (Text S2 and Text S3). The library contains 100 sequences (including the wild-type sequence) with the strength ranging from 0 to 3.559 (relative to the strength of the original sequence), of which 20 sequences are positive mutants (relative strength >1.0) and 79 other sequences are negative mutants (relative strength <1.0).

### Construction and training of ANN predicting models

The initial ANN model was built as a backpropagation model (BP-ANN model) by using Matlab functions provided by Neural Network Toolbox. The model contains three layers, including an input layer, an output layer and a hidden layer. Neuron numbers of the input layer and the output layer were 896 and 1 (determined by the data conversion rule), respectively. For the hidden layer, the number was variable for optimization. The initial weights for all neuron connections were randomly assigned by Matlab functions.

We evaluated the predicting performance by using the sum squared error (*SSE*) between the prediction value 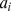 and target strength value 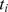 as
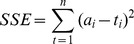
(where *n* is the sequence number of training data set or test data set), and defined prediction error as
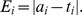



The activation functions of the hidden layer and the output layer were set to be a non-linear sigmoid function ‘logsig’, which was defined as
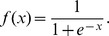



For training of BP-ANNs, a set of example pairs was given as 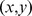, 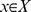 and 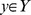, and the aim was to find a function 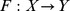 that can match the examples. Here, 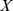 refers to the promoter & RBS sequences and 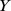 refers to their relative strength. In other words, we wished to infer the mapping relationship between sequence and strength by the samples of training data set. Here, the mapping relationship was a ‘black box’ which can be served as a predicting model for the prediction of test set data. This ‘black box’ may be constructed after training by a set of sample data.

The original sequence data were translated to digital data and served as the input matrix according to the following rules: A = {1, 0, 0, 0}, G = {0, 1, 0, 0}, C = {0, 0, 1, 0}, and T = {0, 0, 0, 1}. For instance, a given sequence ‘ATTGCC’ can be translated to a ‘0-1’ digital series of {1, 0, 0, 0, 0, 0, 0, 1, 0, 0, 0, 1, 0, 1, 0, 0, 0, 0, 1, 0, 0, 0, 1, 0}.

It must be noted that, since the output range of logsig function lies in (0,1), while the target strength can be greater than 1, so it was necessary to normalize the target strength data through dividing by the maximum strength value and then multiplying by this value after simulation.

The goal of *SSE* value for fitting training data was set to be 0.2. The initial weights for all neuron connections were set randomly and automatically by Matlab functions. In addition, ‘traingdx’ was adopted as the learning function; the training epochs and momentum factor were set to be 5,000 and 0.95, respectively.

**Figure 2:**
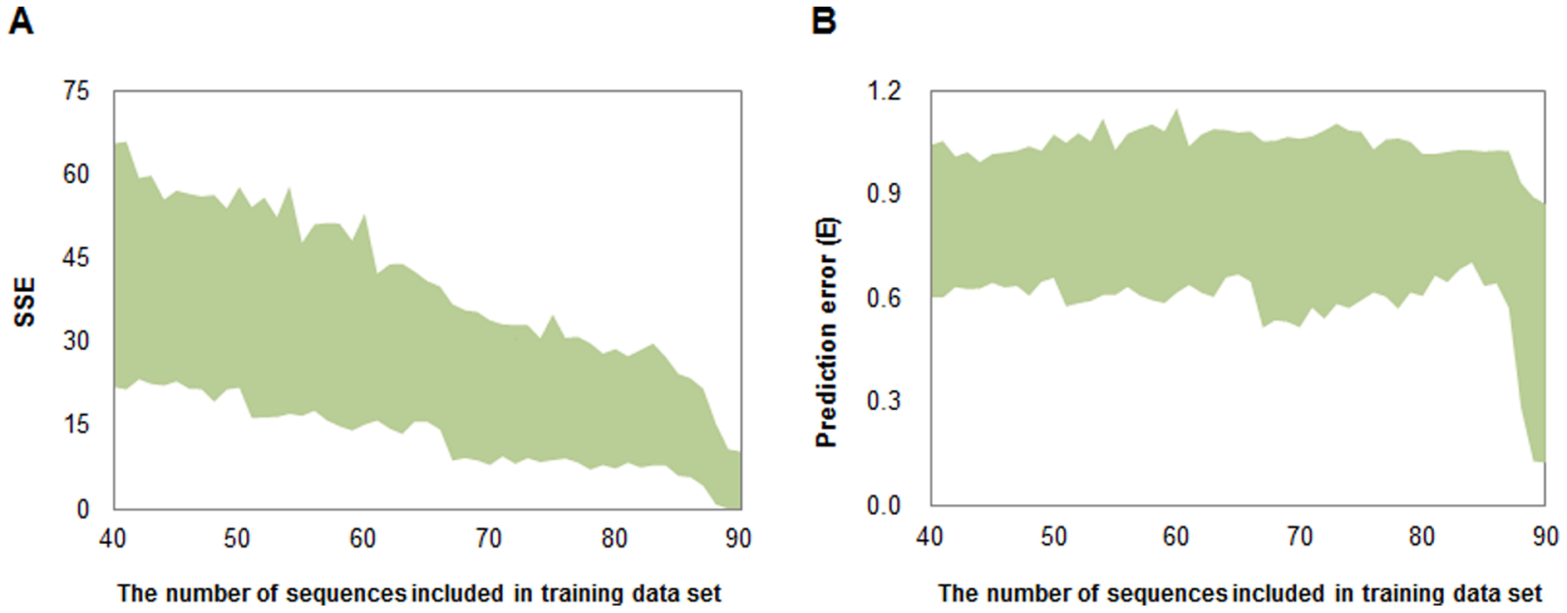
Functional relationship between the prediction performance of ANN models and the scale of training data set. Training data set scale ranges from 40 to 90 sequences. (A) Maximum and minimum *SSE* values of prediction as a function of training data set scale. (B) Maximum and minimum prediction errors as a function of training data set scale.

**Figure 3:**
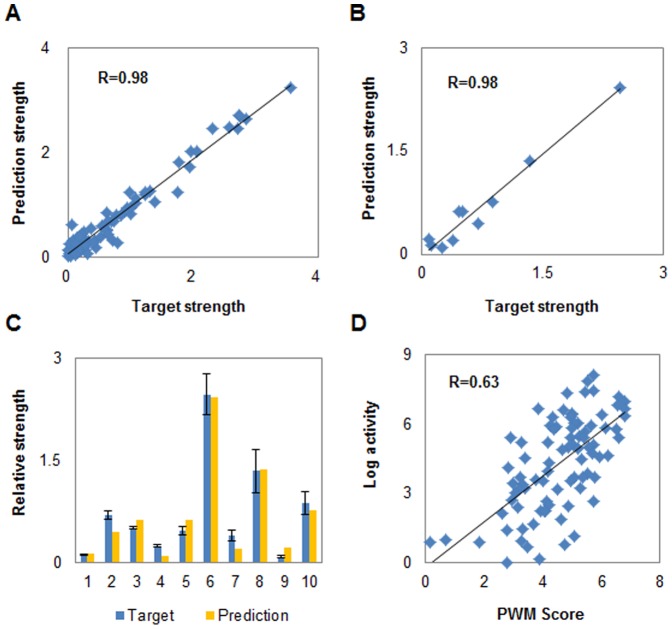
The well trained BP-ANN model NET90_19_576 can finely predict the measured Trc promoter & RBS strengths. (A) The predicted relative strengths of promoter & RBS fit with the measured values using the data of training set. (B) The predicted values fit with the measured values using the data of test set. (C) The comparison results between prediction values and target values (experiment values). (D) The best fitting results of log Trc promoter & RBS activities with their PWM scores.

All 100 sequences in the library were randomly split into two data sets (the training set and the test set) to train and test the ANN prediction models. Considering the effect of the size of training set on the prediction performance, training set was sampled from 40 to 90 sequences (51 situations in total) and each corresponding test set contained the rest sequences. The neuron number of the hidden layer was optimized in a range from 5 to 30, and each trained to generate 1,000 models. Consequently, we obtained 51×26×1,000 = 1,326,000 models. Owing to the random initialization of weights, the trained models have different prediction performance which can be evaluated by their *SSE* and *E* values for prediction of the corresponding test set. Figure 2 describes the overall trends of *SSE* and *E* values as a function of the size of the training data set. Both the maximum and the minimum *SSEs* decline with the increasing size of training data set (Figure 2A), indicating that a certain size of training data set is a requisite for obtaining a model with high predicting performance. For prediction errors *E*, however, both the maximum and the minimum errors do not significantly decline until the size of training set reaches 88 (Figure 2B). Among all generated models, NET90_19_576 (containing 19 hidden layer neurons with a training set's size of 90, see Dataset S1 and Model S1) shows the best performance with the lowest *SSE* of 0.19 and the highest correlation coefficient values of 0.98 for test set prediction. Meanwhile, its correlation coefficient values for fitting the training data set reaches up to 0.98 as well (Figure 3A and 3B), suggesting that this model does not overfit in the process of model training. Besides the high correlation coefficient for the total test set, NET90_19_576 also accurately predicts each element in the test set (Figure 3C). Both the correlation coefficient and the predicting accuracy in our ANN model are significantly improved compared with PLS- [11], PWM- [12] and thermodynamics-based [10] methods. As a comparison, the best result of PWM-based fitting using our data only has an *R* of 0.63 (Figure 3D).

### Quantitative design of promoter & RBS sequences with desired strength

**Figure 4:**
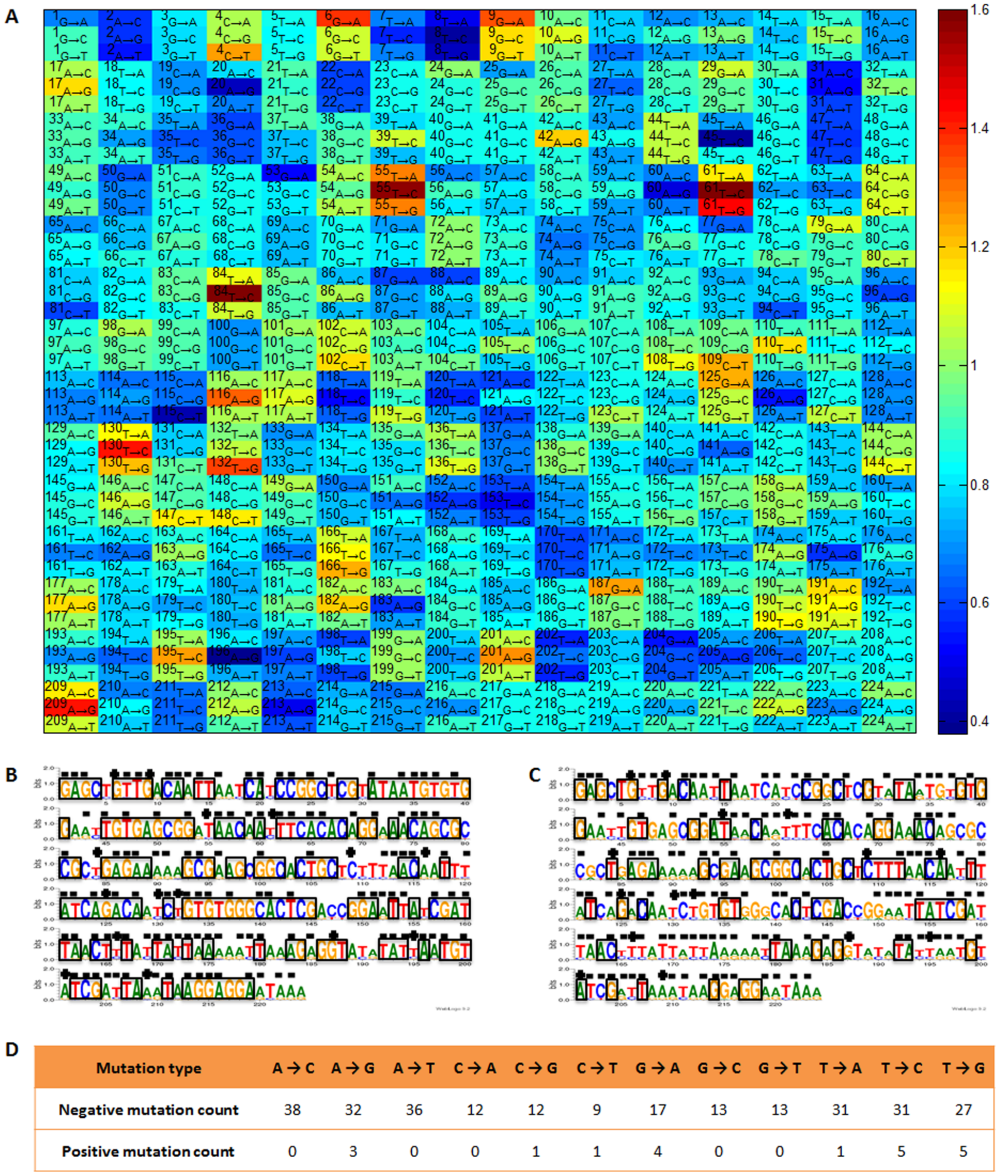
Effect of each single point mutation on sequence strength and sequence conservative analysis. (A) Sequence strength influenced by mutation of each single site. Red indicates positive mutation while blue indicates the negative. Deeper color means more significant change of strength. Each box represents one base in the sequence. Figure in the boxes is the location number of this base, while the subscript indicates that this base is mutated to another one (e.g., A→C means A mutated to C, and T→G means T mutated to G, etc.). (B) Conservative analysis of high activity sequences (strength >1). Bases in the boxes are conservative points. ‘+/−’ indicates this point is predicted to be a positive/negative ‘key-point’. Same as below. (C) Conservative analysis of extremely low activity sequences (strength <0.1). The analysis was performed using online WebLogo Tool (http://weblogo.threeplusone.com/create.cgi). (D) Count of mutation types of the ‘key-points’. Figure in the boxes is the count of negative or positive mutation number.

Owing to the high correlation and accurate prediction performance, the model NET90_19_576 can be effectively developed into a computational platform for quantitative design of novel regulatory parts. Our quantitative design strategy was achieved by consequential *in silico* mutagenesis on native Trc promoter & RBS sequences coupled with rapid strength prediction using NET90_19_576 model. There are two approaches to introduce mutations: i) introduction of random mutagenesis and ii) only introduction mutation of key points (nucleotides significantly affecting the strength). For the first approach, some ‘non-key points’ may be introduced as mutations and increase the calculation time. Besides directly obtaining one (or more) sequence(s), it can randomly generate an *in silico* library in an arbitrary scale (e.g., 10,000 sequences). Hence, we can obtain any desired sequence(s) from this pre-constructed computational library. For the second approach, the effect of single point mutation of wild-type sequence on its strength should be evaluated to determine which points are the ‘key-points’ at first. The strength of mutated sequences may have some correlation with their mutation points. Those sequences with extremely low activity (i.e. m006, m007, m029, etc.) have large amount of mutation points, and most of their functional domains, such as −35 region, −10 region, and RBS region, are destroyed (except for m413, m447, m590, m599). On the contrary, most mutated sequences with high strength have relatively conservative domains. Our results reconfirm that key points significantly affect the sequence strength. We can find out these key points through changing nucleotide one-by-one and use them for designing new element sequences using our computational platform. Figure 4A presents the prediction results of all single point mutations and each point has three mutation types. As a result, 135 points are found significantly impacting the sequence strength, in which 15 points (designated as positive points) can significantly enhance ≥20% of the strength and 120 other points (designated as negative points) can significantly reduce ≥20% of the strength. Sequence logo analysis [33] was also performed to show the most conserved bases among mutations. As a result, most positive impacting points are non-conservative in sequences with high activity (strength >1) (11 of 15, Figure 4B) and conservative in sequences with extremely low activity (strength <0.1) (8 of 15, Figure 4C); in contrast, most negative impacting points are non-conservative in sequences with extremely low activity (90 of 120, Figure 4C) and conservative in sequences with high activity (91 of 120, Figure 4B) as well. That is to say, it is easier to obtain a positive mutation sequence by changing the positive impacting points, or to obtain a negative mutation sequence by changing the negative impacting points. Moreover, most negative points are found to change nucleotide from AT to GC (Figure 4D). It indicates that an increase in GC content may decrease the sequence activity; a higher energy barrier should be overcome for DNA dissociation with a higher GC when transcription and translation initiate.

**Figure 5:**
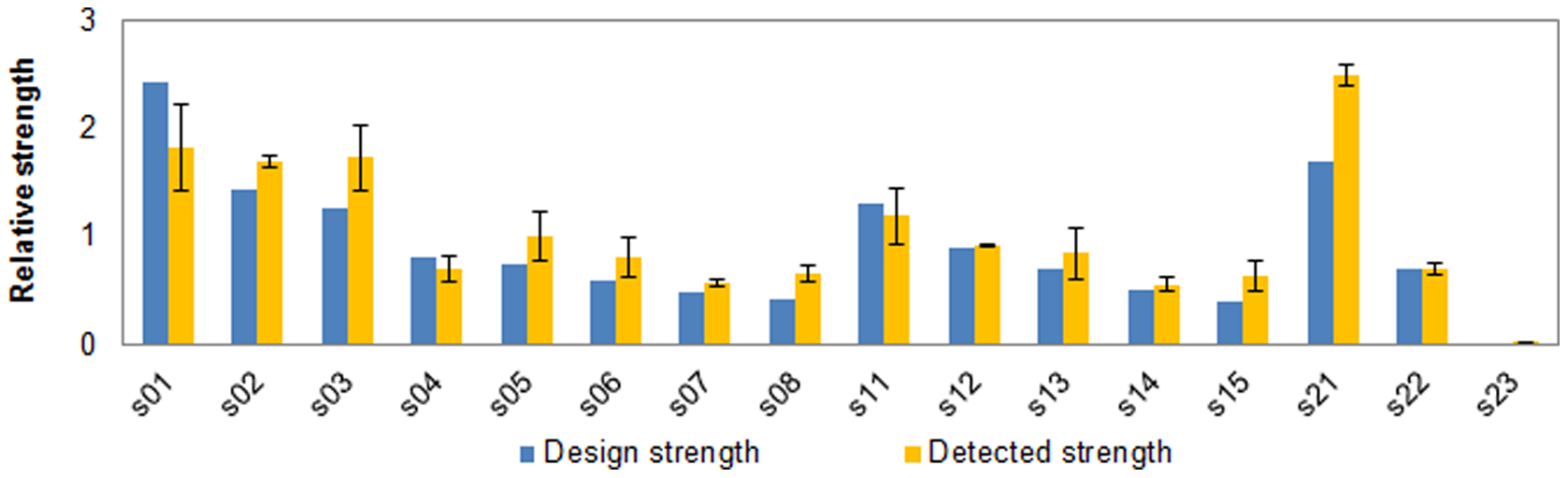
Promoter & RBS sequence design based on ANN prediction model. (A) Sequence with desired strength can be designed by the following strategies: i) 8 out of 10,000 sequences (s01–s08) are randomly selected from an *in silico* Trc promoter & RBS library generated based on ANN predicting model NET90_19_576; ii) sequences (s11–s15) with desired strength can be generated by repeated introduction of random mutations into the wild-type sequence under a certain mutation rate; iii) sequences (s21–s23) with desired strength can be generated by using different combinations of ‘key site’ mutations based on the prediction of NET90_19_576. All designed sequences were synthesized and their strengths were tested and compared with the design strength.

To further verify the effectiveness of our design, sixteen novel Trc promoters & RBS sequences (s01–s08 designed from pre-generated library by approach i, s11–s15 generated from random mutagenesis by approach i, and s21–s23 designed by approach ii) were synthesized *in vitro* and quantified their strengths in strain DH10B (Figure 5, Text S1 and S2). The measured values of all sixteen designed elements show good consistency with our desired strength values, suggesting that both of the above two approaches can achieve quantitative design of novel elements under *in silico* environment.

### Application of designed elements for protein expression and pathway engineering

**Figure 6:**
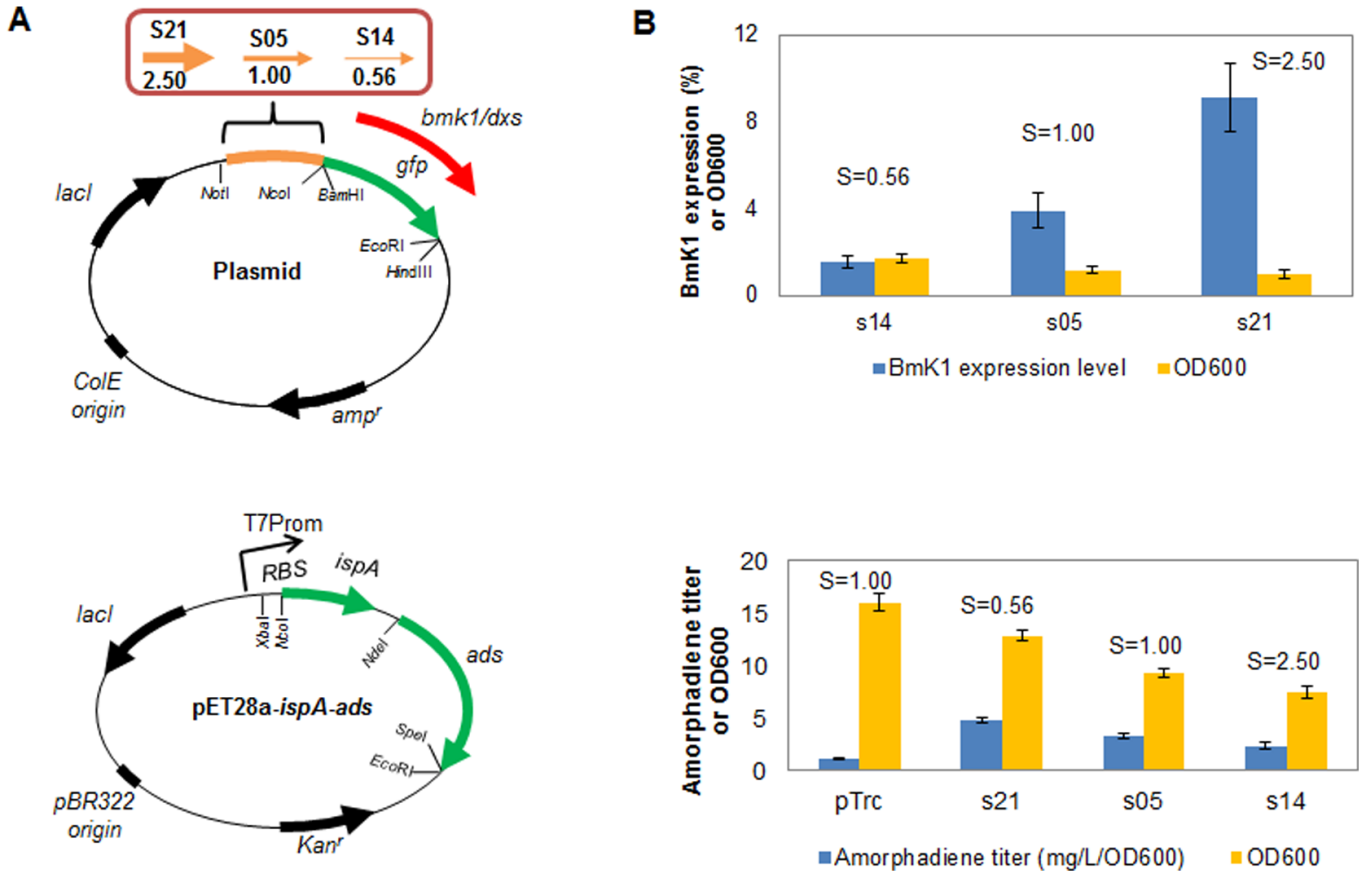
Application of designed elements for peptide BmK1 expression and DXP pathway engineering in *E. coli*. (A) Sketch maps of plasmids for designed elements applications. Plasmids s21-*gfp*, s05-*gfp* and s14-*gfp* contain gene *gfp* between BamHI/EcoRI sites, plasmids s21-*bmk*1, s05-*bmk*1 and s14-*bmk*1 contain gene *bmk*1 between NcoI/HindIII sites, plasmids s21-*dxs*, s05-*dxs* and s14-*dxs* contain gene *dxs* between NcoI/EcoRI sites. (B) Effect of applying designed elements for peptide BmK1 expression and DXP pathway engineering in *E. coli*. The wild-type Trc promoter and RBS (without inserting *dxs* gene) served as the blank control.

The aforementioned work proves that predicting strength of one randomized part and designing a new part are feasible. To further validate the methodology, we need to change the reporter GFP with other metabolic enzymes to test if the designed parts are functionally reliable. Herein we attempted to apply these quantitatively designed regulatory elements in different genetic contexts in strain *E. coli* BL21(DE3), which is protease deficient and suitable for peptide/protein expression. The first case is to optimize heterologous expression of a small peptide BmK1, which is a scorpion toxin secreted by Chinese scorpion *Buthus martensii* Karsch (BmK) and a traditional Chinese medicine for treating ion channelopathies [34], [35]. Like most small peptide toxins, BmK1 is extremely difficult to express in prokaryotic host such as *E. coli* [36], [37]. The strength of promoters has large effects on the production of target protein in surrogate hosts [38], [39], we thus selected three designed elements s14 (strength = 0.56), s05 (strength = 1.0) and s21 (strength = 2.50) to improve BmK1 expression. As shown in Figure 6B, these three elements make great difference for the peptide expression. The expression level of BmK1 was improved from 1.6% (s14) to 9.1% (s21) of total cellular protein with the increase of element strength. This result shows that the strength of our designed elements still agrees with the expression level of the novel genetic context BmKI in BL21(DE3), thus the functional reliability of designed regulatory elements is further verified. In contrast to the expression of GFP without obvious strain growth variations, the growth here was significantly decreased with the accumulation of BmKI, which may probably be due to the cellular toxicity of this small peptide.

The second case is to fine-tune the expression of 1-deoxy-D-xylulose-5-phosphate synthase gene (*dxs*) in *E. coli* BL21(DE3), which has been known to regulate the metabolic flux of deoxy-xylulose phosphate (DXP) pathway [5]. Here we used three designed promoters (s14, s05 and s21) with different strengths to control the expression of *dxs* for improving the supply of isoprenoid precursors. As shown in Figure 6B, the production of sesquiterpene amorphadiene was enhanced with the decrease of element strength. The weakest element s14 achieves the highest yield of amorphadiene (4.82 mg/L/OD_600_). This result agrees with the previous report that fine tuning the expression of rate-limiting enzymes can effectively improve pathway's metabolic flux [5] and further verified the functional reliability of our designed regulatory elements. The strain growth here was also decreased with the enhancement of *dxs* expression and the accumulation of amorphadiene. Both cases demonstrate that our methodology is an effective tool for designing and selecting regulatory element with proper strength for fine-tuning the target gene in metabolic engineering processe. The designed elements with proper strength can achieve both optimized specific product yield and strain growth, which can eventually maximize the process productivity.

## Discussion

Constructing computational models that can precisely predict the strength of a regulatory element and further quantitatively build regulatory elements with desired strength have been a real challenge in gene expression area over decades. Many non-linear or unknown relationships between the sequences of regulatory elements and their strengths are still waiting to be uncovered [7]. We have introduced a methodology for constructing high-precision predicting model based on artificial neural network, which can finely predict the strength of regulatory element by its sequence. Both the high correlation coefficient and the predicting accuracy confirmed that the model is competent for *de novo* quantitative design of desired regulatory elements. The designed elements can be successfully applied in different genetic contexts.

In contrast to the existing prediction models [10], [11], [12], the presented method does not depend on comprehensive understanding of the transcription/translation processes. As most current studies focusing on correlating quantitative characteristics with qualitative information of biological behaviours [40], [41], our method can quantitatively link the strength of regulatory elements with their sequences based on a finely characterized sequence library. The influence of each nucleotide mutation on sequence strength was evaluated and 135 key points were identified in this work, which are very useful for further study of ‘regulatory code’ and *de novo* design of elements. Besides, the methodology can be generalized and applied to construct models for predicting and designing more other promoters and RBSs, and even other regulatory elements like terminators.

Previous studies have confirmed that certain promoters can be identified or predicted based on ANN method [21], [22], [23], [24], [25], [26], [27], [28], but no further effort was reported for quantitative description of their strength. Here, we constructed a finely characterzied Trc promoter & RBS library for sufficient model training and greatly improved the prediction accuracy compared with previous reported methods (PLS-, PWM- and thermodynamics-based) [10], [11], [12]. In addition, we built BP-ANNs using feed-forward as the network structure and Backpropagation (BP) as the training algorithm, mainly due to their rigorous mathematical derivation and proof, well generalization, strong non-linear mapping property, and a wide range of adaptability and effectiveness [15], [16]. The effectiveness and high accuracy of BP-ANNs in constructing strength prediction models has been proven. Here, the initial model only contains one hidden layer, since in theory it can be approximate to a specific function in an arbitrary precision [16]. Adding more hidden layers may enhance the prediction performance but greatly increase the training time. Instead, optimization of the number of hidden layer neurons can also improve the predicting performance [15]. To address the overfitting problem, we tried *SSEs* ranging from 0.001 to 0.5 for model training and found the optimal value of 0.2. The results demonstrate that higher *SSE* makes lower correlation coefficients of fitting for both training data set and test data set. A lower *SSE* generates a higher *R* value of fitting for training data set, but a much lower *R* value of fitting for test data set. In other words, setting a lower *SSE* value of fitting for training data set can easily result in the overfitting problem and bad prediction performance for the test data set. Therefore, setting a suitable *SSE* is important to avoid overfitting problem and achieve the optimal prediction performance.

During the library construction process, we found that large fraction of clones was negative mutants and the probability of picking a positive mutant was less than 0.5%. In contrast, five designed elements with desired strength >1.0 were experimentally verified. These results demonstrate that the present methodology makes great sense for obtaining large amount of elements with different strength without laborious experimental screenings, especially for those stronger elements. But we cannot design a high strong element with a relative strength larger than the maximum value of training data set (3.559), which is limited by the strength range of data samples for model training.

With the rapid development of synthetic biology, quantitative characterization and standardization of regulatory elements will be in general valuable in predicting parts in ever increasing genome sequence data [42]. The presented methodology would help us to easily build high performance prediction and design models using these standardized data in literatures/databases (e.g., the Registry of Standard Biological Parts founded by MIT, http://partsregistry.org) without reconstructing libraries by repeated and laborious experiments. In this framework, our methodology for constructing high prediction performance models and quantitative design of regulatory elements has bright prospects for synthetic biology application.

## Supporting Information

Text S1 **Relative strength value of Trc promoter & RBS elements.**
(DOCX)

Text S2 **Sequences of Trc promoter & RBS elements.**
(DOCX)

Text S3 **Sequence alignment of Trc promoter & RBS elements.**
(PDF)

Dataset S1 **Training data set and test data set for model NET90_19_576.**
(DOCX)

Model S1 **The ANN model NET90_19_576 provided as Matlab format.**
(RAR)
