# Supplementary material for: Quantitative Design of Regulatory Elements Based on High-Precision Strength Prediction Using Artificial Neural Network
Source: PLoS One. 2013 Apr 1;8(4):e60288. doi: 10.1371/journal.pone.0060288 (PMC3613377; doi:10.1371/journal.pone.0060288)
Supplement: Text S1 — Relative strength value of Trc promoter & RBS elements. (DOCX) [file pone.0060288.s001.docx]

**Text S1.** **Relative strength of Trc promoter & RBS elements.**

| No. | Relative strength | Standard deviation | No. | Relative strength | Standard deviation |
| --- | --- | --- | --- | --- | --- |
| m000 | 1.000 | 0.000 | m501 | 1.076 | 0.062 |
| m001 | 1.951 | 0.123 | m505 | 2.469 | 0.298 |
| m003 | 0.699 | 0.089 | m509 | 0.033 | 0.016 |
| m004 | 2.707 | 0.421 | m510 | 0.432 | 0.142 |
| m005 | 0.117 | 0.007 | m514 | 0.174 | 0.023 |
| m006 | 0.013 | 0.003 | m517 | 0.238 | 0.032 |
| m007 | 0.000 | 0.001 | m520 | 0.158 | 0.026 |
| m010 | 3.559 | 0.365 | m521 | 0.734 | 0.142 |
| m014 | 0.070 | 0.009 | m524 | 0.396 | 0.076 |
| m015b | 0.184 | 0.004 | m526 | 1.345 | 0.320 |
| m017 | 0.697 | 0.058 | m534 | 0.154 | 0.049 |
| m018 | 0.760 | 0.088 | m542 | 0.093 | 0.008 |
| m019 | 2.298 | 0.154 | m545 | 0.094 | 0.033 |
| m021 | 0.114 | 0.021 | m546 | 1.224 | 0.063 |
| m024 | 2.569 | 0.465 | m548 | 0.145 | 0.035 |
| m026 | 0.272 | 0.028 | m552 | 0.062 | 0.029 |
| m028 | 1.773 | 0.127 | m565 | 2.848 | 0.285 |
| m029 | 0.036 | 0.018 | m566 | 0.208 | 0.028 |
| m030 | 0.309 | 0.023 | m573 | 0.791 | 0.043 |
| m031 | 0.512 | 0.024 | m580 | 0.014 | 0.002 |
| m054 | 0.239 | 0.024 | m585 | 0.105 | 0.027 |
| m085 | 1.237 | 0.073 | m586 | 0.106 | 0.043 |
| m092 | 0.939 | 0.273 | m587 | 0.606 | 0.133 |
| m150 | 1.299 | 0.318 | m590 | 0.076 | 0.021 |
| m198 | 0.043 | 0.004 | m591 | 0.090 | 0.011 |
| m213 | 0.136 | 0.016 | m599 | 0.001 | 0.000 |
| m232 | 0.617 | 0.141 | m606 | 0.018 | 0.001 |
| m244 | 0.061 | 0.010 | m626 | 0.640 | 0.136 |
| m354 | 0.714 | 0.024 | m629 | 0.395 | 0.069 |
| m360 | 0.209 | 0.109 | m640 | 0.366 | 0.086 |
| m363 | 0.211 | 0.032 | m647 | 0.176 | 0.011 |
| m396 | 0.184 | 0.060 | m659 | 0.910 | 0.178 |
| m412 | 0.065 | 0.003 | m664 | 0.106 | 0.008 |
| m413 | 0.062 | 0.004 | m670 | 0.631 | 0.140 |
| m421 | 0.234 | 0.033 | m675 | 0.880 | 0.166 |
| m424 | 0.128 | 0.021 | m701 | 0.847 | 0.259 |
| m427 | 0.994 | 0.062 | m702 | 1.947 | 0.540 |
| m428 | 0.258 | 0.052 | m705 | 0.987 | 0.276 |
| m430 | 0.249 | 0.019 | m706 | 1.754 | 0.455 |
| m434 | 0.219 | 0.014 | m708 | 2.722 | 0.102 |
| m435 | 0.187 | 0.035 | m709 | 2.056 | 0.367 |
| m441 | 0.156 | 0.043 | m710 | 1.390 | 0.301 |
| m442 | 0.417 | 0.038 | s01 | 1.828 | 0.400 |
| m444 | 0.245 | 0.028 | s02 | 1.699 | 0.062 |
| m445 | 0.327 | 0.111 | s03 | 1.730 | 0.308 |
| m447 | 0.019 | 0.004 | s04 | 0.701 | 0.114 |
| m449 | 0.237 | 0.072 | s05 | 0.999 | 0.226 |
| m454 | 0.509 | 0.103 | s06 | 0.809 | 0.189 |
| m459 | 0.468 | 0.067 | s07 | 0.570 | 0.026 |
| m460 | 0.183 | 0.038 | s08 | 0.657 | 0.072 |
| m463 | 0.610 | 0.385 | s11 | 1.196 | 0.258 |
| m473 | 0.070 | 0.007 | s12 | 0.922 | 0.016 |
| m477 | 0.265 | 0.042 | s13 | 0.848 | 0.241 |
| m479 | 0.067 | 0.008 | s14 | 0.560 | 0.060 |
| m483 | 0.528 | 0.104 | s15 | 0.640 | 0.136 |
| m484 | 2.740 | 0.463 | s21 | 2.501 | 0.093 |
| m489 | 0.167 | 0.016 | s22 | 0.705 | 0.052 |
| m491 | 1.071 | 0.076 | s23 | 0.025 | 0.001 |
