# Supplementary material for: Quantitative Design of Regulatory Elements Based on High-Precision Strength Prediction Using Artificial Neural Network
Source: PLoS One. 2013 Apr 1;8(4):e60288. doi: 10.1371/journal.pone.0060288 (PMC3613377; doi:10.1371/journal.pone.0060288)
Supplement: Text S2 — Sequences of Trc promoter & RBS elements. (DOCX) [file pone.0060288.s002.docx]

### Text S2. Sequences of Trc promoter & RBS elements.


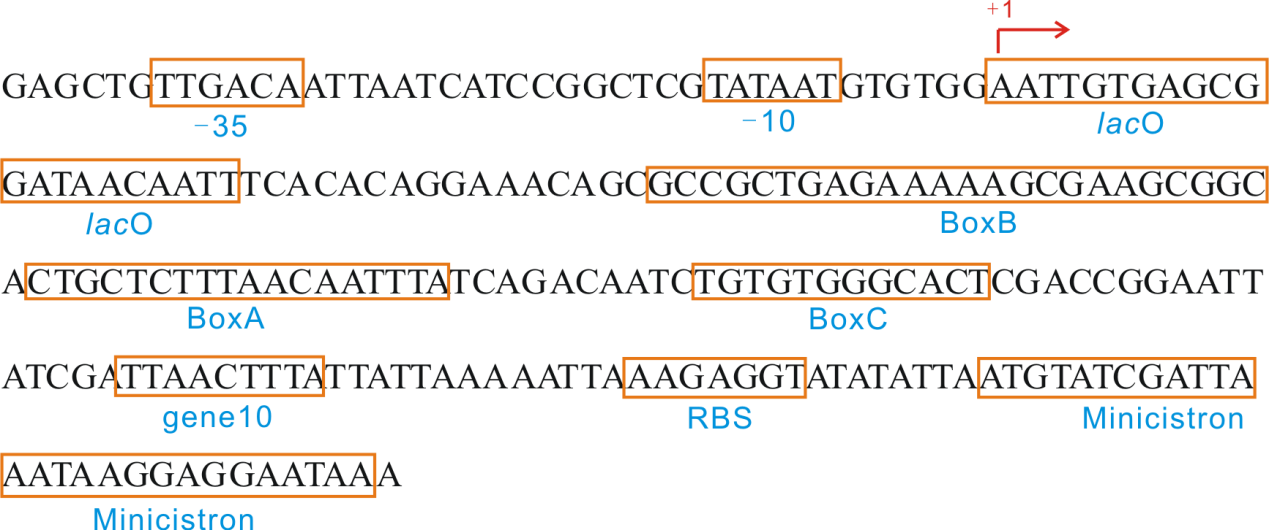


***Notes*:**

-35 Region：TTGACA, (76 sequences conserved)

-10 Region：TATAAT, (68 sequences conserved)

*lacO*：AATTGTGAGCGGATAACAATT, (32 sequences conserved)

*gene10*：TTAACTTTA, (45 sequences conserved)

Minicistron：ATGTATCGATTAAATAAGGAGGAATAA, (16 sequences conserved)

RBS：AAGAGGT, (65 sequences conserved)

**Sequences of Trc promoter/RBS library:**

m000: strength = 1.000

GAGCTGTTGACAATTAATCATCCGGCTCGTATAATGTGTGGAATTGTGAGCGGATAACAATTTCACACAGGAAACAGCGCCGCTGAGAAAAAGCGAAGCGGCACTGCTCTTTAACAATTTATCAGACAATCTGTGTGGGCACTCGACCGGAATTATCGATTAACTTTATTATTAAAAATTAAAGAGGTATATATTAATGTATCGATTAAATAAGGAGGAATAAA

m001: strength = 1.951

GAGCTGTTGACAATTAATCACCCGGCTCGTATAATGTGTGGAATTGTGAGCGGATAACAACTTCACACAGGAAACAGCGCCGCTGAGAAAAAGCGAAGCGGCACTGCTTTTTAACAATTTATCAGACAATCTGTGTGGGCACTCGACCGGAATTACCGATTAACTTTATTATTAAAAATTAAAGAGGTATATATTAATGTATCGATTAAATAAGGAGGAATAAA

m003: strength = 0.699

GAGCTGTTGACAATTAATCATCCGGCTCGTATAATGTGTGGAGTTGTGAGCGGATAACGACTTCCCACAGGAAACAGCACCGCTGAAGAAAGGCGAGGCGGCGCTGCCCTTTAACGACTTACCAAACAGTCTGTACGGGCACTCGACCGGAATTATCGATTAACTCTACTGTCAAAAGTTAAGGGGGTATATATTGATGTATCGACTAAGCAAGGAGGGGTAGC

m004: strength = 2.707

GAGCTGTTGACAATTGGTCATCCGGCTCGTATAATGTGTGGAACTGTGAGCGGATAACGATCTCACACAGGAAGCAGCGCCGCTGGGGAAAAGCGAAGCGGCACTGCTCTTTAACGATTTATCAAACGACCGGTGTGGGCACTCGGCCGGAATTACCGATTAGCTCTATTACCAAAAATTAAAGAGGTATGTATTAATGTACCGACTAAGTAAGGAGGAACAGA

m005: strength = 0.117

GAGCCGCTGGCAACTAGTCATCCGGCTCGTGTAATGTGTGGAATTGTGAGCGGGTAACAGTTTCACACAGGGAACGGCGCCGTTGAGGAAAAACGGAGCGGCACTGCTCTTTAACAACTTATCAGACAATCCGTGTGGGCACTCGACCGGAATTATCGGTTAACTTTACTATCAAAAATTAGGGAGGTATATATTAGTGTATCGATTGAATAAGGGGGAATAGA

m006: strength = 0.013

GGGCTGCCGGCAATTAATCACCCGACTTGTGTAGTGGGCGGAGTCGTGAGCGGATAGCAACCTCACGTAGGAGACGGCATCGCTGAAAAGGGGCGAAGCGGCACTGCTCTTTAACAGCTCATTAGGCAACCTGCGTAAACACTCGACCGGGGTTATCGACTAACCCTATTGCCAGGGACTGAAGAGATGCGCACTAGTGTATCGGTTAAGTAAGGAGGAATAAA

m007: strength = 0.000

GGGCTGTTGACAATTAATTATCCGACTCGCGCAAGGCGTGGAATTGTGAGCGGATTACGGCCTCACACAGGAAACAGCGCCGCTGGGGAGGAGTGAGGCGGCACTACTCTTTAACAATTTATCAGGCAATCCGTGTAGGCACCCGATCGGAATTATCGACCAACCCCATTACTAAAGATTAAGGGGGTATATACTAATGTATCGATTAAGTAGGGAGGAATAAA

m010: strength = 3.559

GAGTTGTTGACAGTTAATCATCCGGCCCGTACAGTGTGTGGGACTGTGAGCGGACAGCAACTTTACACAGGAAACAGCGCCGCTGAGAGAAAGCGAAGCAGCACTGCTCCTTAACAATTTATCAGACAGTTTGTGTGGGCACTCGACTAGAATTATCGATTAACTTTATTATTAAAAATTAAAGAGGTATATATTAATGTATCGATTAAATAAGGAGGAATAAA

m014: strength = 0.070

AAGCTGTTGACAATTAATCACCCGGCTCGTATAGTGTGTGGAATTGTGAACGGATCACAGTCTCACACAGGAGACAGCGCCACCGAGAGAAAGCGAAGCGGCACTGCTCTTTAACAACTTATCAGACAAGCTGTGTGGGCACCCGACCGAAATTATCGACTAACTCCATTATTAAAAATTAAGGGGGTATATACTAAGGTACCGATTAAATGAGGGGGAACAAA

m015b: strength = 0.184

GGGCTGTTGACAATTAACCATTCGGCTCGTGTAATGTGTGGAACTGTGAGCGGATAACAGTTTCACACAGGAAACAGCACCGCTGAGGAAAAGCGAAGCGGCATTGCTCTTTAGCAATTTATCAGACAATCTGCGTGGGCACTCGACCGGAATTATCGGTTAACTTTACTATTAGGGACTAAAGAGGTATGTATTAGTGTATCGATTAAATAAGGAGGAATCAA

m017: strength = 0.697

GCGCTGTTGACAAGTGATCATCCGATTCGTATAATGTGTGGAATTGTGAGCGGATAACAATTTTACACAGGCAATAGTGCCGCTGAGAAAAAGCGAAGCGGCACTGTTCCTCAACAACTTATCAGACAGCCCGTGTGGGCACTCGATCGGAGTTATCGGTCAACTCTATCATTAAAGACCAAAGGGGTATATATTAATGTATCGACTAAGCAAGGGGGGCTAGA

m018: strength = 0.760

GAGCTGTTGGCAACTGATCATCCGGCTTGTATAATGTGTGGAGTGGTGAGCGGATAATAACTCCGCACAGGAGACAGCGCCGCTGAGGAAGAGCGAGGCGGCACTGCCCCTTAACGATCTATCAGACAATCTGCGTGGGCACTCGACCGGGATTGCCGACTAACTTTATTACTAGAAGTTAAAGAGGTGTATACTAATGTATCGGTTGAATGAGGGGGAATAAA

m019: strength = 2.298

GAACTGTTGACAATTAATCATCCGGCTCGCATAATGTGTGGGACCGTGAGCGGGTAACAACTCCACACAGGGAACAGCGCCGCCGAGAAGGGGCGAAGCGGCACCGCTCTTTAACAGTTTATCAGACAATTTGTGTGGGTACTCAACCGGAATTACCAATTAACCTCGTTACTGAGGATTAAAGGGGTGTAGATCAGTGTATCGATTAAATAAGGAGGAATAAA

m021: strength = 0.114

AAGTTGTTGATAGTTAACCACCCGGCTCGTATAATGCGTGGAGTTGTGAGCGGATAACAATCTCACACAGGGAACAGCGCCGCTGAGAAGGAACGAAGCAGCACCGCTCCCTAACAGTTCATCAGGCGACCTGCGTGGGCGCCCGACCGAGACTGTCGGTTAACCCTACTATCAAGGACCAAAGGGGTATATATTGGTACATCGACTGAGTAAGGAGGAGTAAG

m024: strength = 2.569

GGGCTGTTGACAGTTAACCATCCGGTTCGTATAATGCGTGGAGTTGTGAGCGGATAACGATTTCACACAGGGAACGGCGCCGCCGAGAGAAAGCGGAGCGGTACTGCTCCTCAACAATTCATCAGACGATCTGTGTGGGCACTCGACTGGAGTTATCGATTAGCTCTACTATTAAGGATTAGCGGGGTATATGCTAATGTGTCGATTAAATAAGGAGGGATGGG

m026: strength = 0.272

GAGCTGTCGACAATTAACCGTCCGGCCCGTATAATGCGTGGAACTGTGGGCGGATGACAATTCCGCACAGGAAACAGTGCCGCTGGGAAGAAGCGAGGCGGCACTGCCCTTTGACAGTCTATCAGACAATCTGCGTGGGCACTCGACCGGAATTATCGATTAACCTCACTATTAGAAATTGGAGGGACATATATCACTGTACCGACTAAACAAGGAGGAATCAG

m028: strength = 1.773

GAGCTGCTGGCAATCAATCATCCGGCTCGTATAACGTGTGGAACTGTGAGTGGATAACAATTTCACACAGGAAACAGCGCCGCCGAGAAGAAGCGAGGCGGCGCTGCTCCCTGACAATTTATCAGACAATCTGTGTGGGCACTCGATCGGAGTTATCGATTAACTCTACTATTGAAAGTCGAGGGGGTATACATTAATGCATCGGTTAGATAAGGGGGAATAAA

m029: strength = 0.036

GAGCTGTCGACAACTAGTCATCCGGCTCGTATAGTGTGTGGAATTGTGAGCGGATAGCAGTTTCACACGGGAAACAGCGCCACTAAGAAGAGGCGGGGCGGCACTGCTCCTTAGCAACTTATCAGGCAGTTTGTGTGGGCGCCCGACTGGGACTATCGGTTGGCCCTGCTATTCAAGGCTAAAGGGACGCGTACCAATGCATCGGTTGAATAAGGGGGAATAAA

m030: strength = 0.309

GAGCTGTTGACAACTAATCATCCGACCCGTATAACATGCGGAATTGTGAGCGGACAACAGTTTCACACGGGGGGTGGCGCCGTCGAGGAGAGATGCAGCGGCACTGCTCTTTAGCAGCCTATTAGGCAGTCTGTATGGGCACCCGACCGGGATTACCGATTAGCCTCACTATTAAGAGCTAAAGGGGTGTGTACTAACGTACCGATCAAATAAGAAGGAATAAA

m031: strength = 0.512

GGGCTGTGGACAGTTGATCATCCGGCTCGTATAATGTGTGGAATTGTGAGCGGATAACAGCTTCGCACAGGAAACAGCGCCGCTGAGGGAAAGCGAAACGGCACTGCCCCTTAGCAATCTATCAGACAATCTGCGTGGACACCCGACCGGAGCTATCAGTTAACTCTATTATCAGAAATTAGAGAGGTATACATTCATGTATCGATTGAATAAGGAGGAATGAG

m054: strength = 0.239

GAGCTGTTGACAGTTAATCGTCCGGCTCGTGTAATGTGTGGAATTGTGAGCGGATAACAATCTCACACAGGAAACAGCGCCGCTGAGAAAAAGCGGAGCGGCACTGCTCCTTAACAATTTATCAGACAGTCTGTGTGGGCACTCGACCGGAATCATCGATTAACTCTATTATTAGAAATTAAAGAGGTATATATCAATGTATCGATTAAGTAAGGAGGAATGAA

m085: strength = 1.237

GAGCTGTTGGCAATTAACCATCCGGCTCGTATAATGTGTGGAATTGTGAGCGGGTAATAACTTCACACAGGAAACAGCGCCGCTGAGAAGAAGCGAAGCGGCACTGCTCTTTAGCAGCTTATCAGACAATCTGTGTGGGCACTCGGCCGGAGTTATCGATTAACTTTACTATTAACAGTTAAAGAGGTATATATTAATGTATCGATTAAATAAGGAGGAATAAG

m092: strength = 0.939

GAGCTGTTGACGGTTAATTATCCGGCTCGTATAATGTGTGGAATTGTGAGCGGGTAACAATTTCACACAGGGAACAGCGCCGCTGAGCGGAAGCGAAGCGGCACTGTTCTTTGACAATCTGTCAGACGATCTGCGTGGGCACTTGACCGGAATTATCGACTAGCTTTGTTGTTAGAAATTAAAGAGGCATATATTAATGTACCGATTAAATAAGGAGGAATAAA

m150: strength = 1.299

AAGCTGTTGACGATTGATCACCCGGCTCGCATAATGTGTGGAGTTGTGAGCGGGTAACAACTCCACACAGGGAACAGCGCCGCTGAGAAAAAGCGGAGCGGCACTGCTCTTTGACGATCCATCGGGCAGCCCGTGTGGGCACTCGATCGGAGTTACCGATTAACTTTATTATTAAAAATTAAAGAGGTATATATTAATGTATCGATTAAATAAGGAGGAACAAA

m198: strength = 0.043

GAGCTGCTGACAATTAATCACCCGGCTCGTATAATGCGCGGAACTGTGAGCGGATAGCGGTTCCACACAGGAAACAGTGCCGCTGAGAAAAAGCGAAGCGGCGCTGCTCTTTAACAATTTATCGGGCAATCTGTGTGGGCACTCGACCGGAATTACCGATCAACTTTGTCATCAAGAGTTAAGGGGGTGCATATTGATGTATCGATTAAACAAGGGGGGATAAG

m213: strength = 0.136

gagctgttgacaattaatcatccggctcgtctaatgtggggaattgtgagcggataacaatttcacacaggaaacagcgccgctgagaacaagcgaagcggcactgctctttcacaatttatcagacaatctgtgtgggcactcgaccggaattatcgattaactttattattcaaaattaacgaggtatatattaatgtatcgattaaataaggaggaataaa

m232: strength = 0.617

gagctgttgacaattaatcatccggctcgtataatgtgtggaattgtgagcggataacaatttcacacaggaaacagcgccgctgagaaaaagcgaagcggcactgctctttagcaatttatcagacaatctgtgtgggcactcgaccggaattatcgattaactttattattaaaaattaaagaggtatatattaatgtatcgattaaatcaggaggaataaa

m244: strength = 0.061

GAGCTGTTGACAATTAATCATCCGGCTCGAATAATGTGTGGAATTGTGAGCGGATACCAATTTCACACAGGAAACAGCGCCGCTGAGAAAAAGCGAAGCGGCACTGCTCTTTAACAATTGATCAGACAATCTGTGTGGGCACTCGACCGGAATTATCGATTAACTTTATTCTTAAAAATTAAAGAGGTATATATGAATGTATCGATTAAATAAGGAGGAATAAA

m354: strength = 0.714

GAGCTGTTGACAATTAATCGTCCGGCTCGTATAGTGTGTGGAATTGTGAGCGGATAACAACTTCACGCAGGAAACAGCGCCGCTGAGAAAGAGCGAGGCGGCGCTGCCCTTTGACAATTTGCCAGACAATCTGCGTGGGCACTCGACCGGAATTATCGATTAACTTTACTATTAAAAATTAAAGAGGTATATACTAATGTACCGACTAGACAAGGAGGAGTAGA

m360: strength = 0.209

GAGTTGTTGACAATTAATCATCCGGCTCGTATGATGCGTGGAGTTGTGAGCGGGTAACAACTCCACACGGGAAACAGCGCCGCTGAGAGAAAGCGAAACGACACTGTTCTTTAACAGTTCATCAGACAATCTGCGTGGGCACTCGACCGGGACTATCGATTAACTTTATTGTTAAAAGTTAAGGAGGTATATATTGATGTATCGATCAAATAAGGAGGAATAAA

m363: strength = 0.211

GAGCTGTTGACAATTAATCATCCGGCCCGCGTAATATGTGGAATTGTGAGCGGATAACAGTTCCACACAGGAAACAGCGCCGTTGAGAAGAAGCGAAGCGGCACTGCCCTTTAGCAATTTATCAGACAATCTGTGTGGGCACCCGACCGGAATTATCGAGTAACTTTGTTACTAAAAATTAAAGGGGTAGACATTAATGTATCGATCAAATGAGGGGGAGTAAA

m396: strength = 0.184

GAGCTGTTGACAATTAATCATCCGGCTCGTACAATGCGTGGAATTGTGAGCGGATAACAATTTCACACAGGAAACAGCGCTGCTGAGAAAAAGCGAAACGGCACTGCTCTTCAACAGTTTATCAGACAATCTGTGTGGGCACTCGACCGGAATTATCGATTAACTTTATTATTAAAAACTAAAGAGGTATATACTAATGTACCGGTTAAACAAGGAGGAATAAA

m412: strength = 0.065

GAGCTGTTGGCAATTAATCATCCGGCTCGTCTAATGCGTGGAATTGTGAGCGGACAACAATTTCACACAGGAAACAGCGCCGCTGAGAAAAGGCGAAGCGGCACTGCTCTTTAACAATTTGTTAGACAATCTGCGTGGGCACTCGACCGAAATTGTCGATTAACTTTATTATTAAAAACTAAAGAGGTATATATTAGTGTATCGATTAAATAGGAAGGAGTAAG

m413: strength = 0.062

GAGCTGTTGACAGTTAATCATCCGGCTCGTATAATGTGTGGAATCGTGAGCGGATAACAGTTTCACACAGGAAACAGTGCCGCTGAGAAAAGGCGAAGCGGCGCTGCCCTTTAACAATTTATCAGACAATCTGTGTGGGCACTCGACCGGAGTCATCGATTAACGTTATTATCAGAAATTAAAGAGGTATATGTCAGTGTATCGACTAAATAAGGAGGAATAGA

m421: strength = 0.234

GAGCTGCTGACAATTAGTCATCCGGCTCGTATAATGTGTGGAATTGTGAGCGGATAACAGTTTCACACAGGAGGCAGCGCCGCTGAGAAAAAGCGAGGCGGCACTGCTCTTTCACAATTTATCAGACAATCTGTGTGGGCACTCGACCGGAGTTATCGACTAACCTTATTAGTAGAGATTGAAGAGGTGTATATTAATGTATCGGTTAAATAAGGAGGAATAGA

m424: strength = 0.128

GAGCTGTTGACAGCTAATCATCCGACTCGTATAATGTGTGGAATCGTGAGCGGATAACAATTTCACACAGGAAACAGCGCCGCTGAGAAAAAGCGAAGCGGCACTGCCCTTTAACAATTTATCAGACAATCTGTGCGGGCACTCGACCGGAACTATCGATTGACTTTATTATTAAGAGTCAAGGGGGTGTATGTTAGTGTATCGATTAAGTAAGGAGGAATAAA

m427: strength = 0.994

GAGCTGTTGACAATTAATCATCCAGCTCGTACGATGTGTGGGATTGTGAGCGGATAACAATTCCACACAGGAAACGGCGCCGCTGAGAAAAAGTGGAGTGGCGCTGCTCTTTAACAATTCATCAGACAACCTGTGTGGGCACCCGACCGGAATTGTCGGTTAGTTTTACTATTAAAAATTAACGAGGTATATACTAATGCATCGATCAAATAAGGAGGAGTAAA

m428: strength = 0.258

GAGCCGTTGACAGTTAATCATCCGGCTCGTGTAATGTGTGGAGTCGTGAGCGGATAACAATTTCACACAGGAAACAATGCCGCTGAGAAAAAGCGAAGCGGCACTGCTCTTTAACAATCTATTAGACAGTCCGTGTGGGCACTCGACCGGAATTATCGACTGACTTTATTATTAGAGACTAGAGAGGTATACACTAATGTACCGATTAAATAAGGGGGGACAAA

m430: strength = 0.249

AAGCTGCCGATAACTAACCATCCGGCTCGTATAATGTGTGGAGTTGTGAACGGATAACAATTTCACACAGAGAACAACGCCGCGGAGAAGGAGCAAAGCGGCACTGCTCTTTAACAACTTGTCAGACAGTCTGGGTGGGCACTCGACCGGAGTTGTCGATTAGCTTTATTATCAAAGACTAAAGAGGTATACATTAACGTATCGACTAAATGAGGAGGAATAAG

m434: strength = 0.219

GAGCTGCTGACAATTAATCATCCGGCTCGTATAATGCGTGGAACTGTGAGCGGACAACAATTTCACACAGGAGACAACGCCGCTGAGAGAAAGCGGAGCGGCACTGCTCCTTGACAATTTATCGGACAATCTGTGTGGGCACTCGACCGGAATTATCGATTAACTTTATTACTAAAAGTTAAGGAGGTATATATTAATGTATCGATTAAATAAGGAGGAATAGA

m435: strength = 0.187

GAGCCGCTGATGATTAATCGTCCGACTCGTACAGTGTGTGGAATTGTGAACGGATAACAATTTCGCACAGGAAACAGCGCCACTGAGAAAAAGCAAGGCGGCACTGCTCTTTAACAATTTATCAGACAATCTGTGTGGGCGCTCGACCGGAACTATCGATTAGCTTTACTATCCAAAATCGAAGAGATACATACTAATGCATCGACTAAATAAGGGGGGATAAA

m441: strength = 0.156

GAGCTGTTGGCAGCTAGTCATCCGGCTTGTATAATGTGTGGAATTGTGAGCGGATAACAATTCCACACAGGAAGCAGCGCCGCTGAGAGAGAGCGAAGCGGCACTGCTCCCTAACAGTCTGTCAGATAATCTGCGTGGGCACCCGACCGGAGCCATCGATTAACTCTATTATTGAAAACTGAAGAGGTATATACTGATGTACCGACTGAATAGGGAGGGATAGA

m442: strength = 0.417

GAGCTGTTGGCAATTAATCATCCGGCTCGTACAACGTGTGGAATTGCGGGCGGATAACAGTTTCACACGGGAAACAGCGCCGCTGAGAAAGAGCGAAGCGGCGCTGCTCTTTAACAATTTATCAGACAGTCTGCGTGGGCACTCGACCGGAGTCATCGATTAACTTTATTATTAAAGACTGAAGAGGTGTATATTAATGTATCGATTAAATAAGGAGGAATAAA

m444: strength = 0.245

GGGTTGTTGACGACTAATCATCCGGCTCGTGTAATATGTGGGATCGTGAGCGGATGACAATCTCGCACAGGAAGCAACGCCGCCGAGAAGAAGCGGAGCGGCACTGCTCCTTAACGACTTATCAGACAATCTGTGTGGGCACTCGACCGGAGTCATCGATCAGCTTCATCATTAAAAATTAAAGAGGCGCATATTAATGTATCGATTAAGTAAGGGGGAATAAA

m445: strength = 0.327

GGGCTGTTGACAATCAATCACCCAGTTCGTACGATGTGTGGAATTGTGAGCGGGTGACAATTTCACACGGGAAACAGCGCCGCTGAGGAAAAGCGAAGCGGCACTGCTCTTTAACAATCTATCAGACAATCTGTGTGGGCACTCGACCGGGGTCATCGATTAACTTTACTATTGAAAATTAAAGAGGTGTACATTGGTGCATCGATTAGATGAGGAGGAGTAGG

m447: strength = 0.019

GAGCTGTTGACAACTGATCACCCGGCTCGTATAATGCGTGGAGCTGCGAGCGGATAACAATTTCACACAGGAGACAGCGCCACTGAGAAAAAACGAAGCGGCACTGCTCTCTAGCAGTCTATCAGGCAATCTGTGTGGACGCTCGACCGGGATTATCGATTAACTTTATTATTAGGGGTTAAAGAGGTACATATTAACGTATCGGTTAAATAAGGAGGGATAGA

m449: strength = 0.237

GAGCTGTTGACAATTAATCATCCGGCTCGTATAGTGTGTGGAGTTGTGAGCGGATGACAGTCTCGCACAGGAAACAGCGCCGCTGGGAAAAAGCGAAGCGGCACTGCTCTTTAACAATTTATCAGACGATCTGTGTGGGCGCCCGACCGGAATCATCGGTTAACTTTATTACTAAAAACTAGAGAGGTATATATTAATGTATCGATTAAATAAGGAGGAATGAA

m454: strength = 0.509

GAGCTGCTGGCAGTTGATCGTCCGGCTTGTATAATGTGTGGAGTTGTGAGCGGATAACAATCTCACGCAGGGAACAGCGCCGCTGAGGAGAAGCGAAGCAACACTGCTCTTTAACGATTTATCAGACAATCTGTGTGGGCGCTCGGCCGGAATTATCGATCAACTTTACTATTAAGAATTAAAGGGGTATATATTAATGTATCGACTAAATAAGGAGGAGTAAG

m459: strength = 0.468

GAGCTGTTGACAGTTAGTCATCCGGCTCGTATAATGTGTGGGGCTGCGAGCGGACAACAGTTTCGCACAGGAAACAGCGCCGCTGAGAGAAAGCGAAGCAGCACTGCTCTCTAACAGTTTATCAGACAATCTGTGTGGGCACTCGACCGGGGTTATCGGTTAACTTTATCATTAAAAATTGAGGGGGTATGTACTAATGTGTCGGCTAAATAAGGAGGGATGAG

m460: strength = 0.183

GAGCTGTTGACAATTAATCATCCGGCTCGTATAGCGTGTGGAATTGTGAGCGGATAATAATTCCACACAGAAAACCGCGCCGCTGAGGAAAAACGAAGTGGCGCTGCTCTTTAGCAATTTATCGGACAACCTGTGTGGGCACTCGACCGGGATTATCAATTAGCTTTATCATTAAGAATTGAAGAGGTGTATACTCGTGTGTCGATTAGATGAGGAGGAGTAAG

m463: strength = 0.610

AAGCTGTTGACAATTAACCATCCGGCTTGTATGATGTGCGGAATTGTGAGCGGATAACAATTCCACACAGGAGATAGCGCCGCTGAGGAAAAGCGGAGCGGCACTGCCCTTTAGCAATTTGTCAGACAACCTGTGTGGGCACTCGACCGGAGTTGTCGATTAACTTTATTACTGAAGATTAGAGAGGTATATATTAATGTACCGGTTAAACAAGGAGGAATAAA

m473: strength = 0.070

GAGCTGCTGACAATTAATCATCCGGCCCGTATAATGTGTGGGACCGTGGGCGGATAGCAGTTTTACACAGGAAACAACGCCGTTAAGAAAGAGCGAAGCGGCGCTGCGCTTTAACAACTTGTCGGACAATCCGTGTAGACACTCGACCGGAGTTATCGATTAACCTTATTGTTAAGAACTAAAGAGGTGTGTATTAATGTATCGACTAAGTAAGGAGGAATAAA

m477: strength = 0.265

GAGCTGCTGACTATTAACCACCCGGCTCGTATAATGTGTGGAATTGTGAGCGGATAACAATTTCACACAGGAAACAGCGCCGCTGAGAAAAAGCGAAGCGGCACTGCTCTTTAACAACTTATCAGACAATCTACGTGGGCACTCGACCGGAGTTATCGATTAACTTTGTTGTTAAAAATTAAAGAGGTATACATTAATGTATCGATTAAATGAGGAGGAATAAA

m479: strength = 0.067

GAGCTGCTGACAATTAATCATCCGGCCCGTATAATGTGTGGGACCGTGGGCGGATAGCAGTTTTACACAGGAAACAACGCCGTTAAGAAAGAGCGAAGCGGCGCTGCGCTTTAACAACTTGTCGGACAATCCGTGTAGACACTCGACCGGAGTTATCGATTAACCTTATTGTTAAGAACTAAAGAGGTGTGTATTAATGTATCGACTAAGTAAGGAGGAATAAA

m483: strength = 0.528

GAGCTGTTGACAACTAACCATCCGGCTCGTATAACGTGTGGAATTGTGAGCGGATAACAACTTCACGCAGGGGATAGCGCCGCTGAGAAAAGGCGAAGCGGCACTGCTCTTTAACAATTTATCAGACAATCTGTGCGGGCACTCGACCGGAGTTATCGATTAACTTTATTATCAAAAGCTAAAGAGGTATATATTAATGCATCGATTAAGTAAGGGGGAATAAA

m484: strength = 2.740

GAGCTATTGACAATTAATCATCCGGCTCGTATAATGTGTGGAATTGTGAGCGGATAACAATTTCGCACAGGAAACAGCGCCGCTGAGAAAAAGCGAAGCGGCACTGCTCCTTAACAATTTACCAGACAATCCGTGTGGGCACTCGACCGGAATTATCGGTTAACTTTACTATTAAAAGTTAAAGAGGTATGTATCAATGTATCGATTAAATAAGGAGGAATGAA

m489: strength = 0.167

GGGCTGTTGACGATTAATCATCCGGCTCGTACAATGCGTGGAACCGTGAGCGGATAACAACTTCACACAGGAAGCAGCGCCGCTGAGGAAAAGCGAAGCAGCATTGCTCTTTAGCAACTCATCAGACAATCTGTATGGACACTCGACCGGGATTATCGATTGGCCTCGCTATTAAGAATTAAAGAGGTGTACACTAATGCATCGATTGGATAAGGAGGAATGGA

m491: strength = 1.071

GAGCTGTTGACAGTTAATCACCCGGCCCATATAATGTGTGGAATTGTGAGCGGATGACAACTTCACACGGGGAACAGCGTCGCTGAGGAAGAGCGAGGTGGCACTGCTCTTTAGCAATTTATCAGACAGTCTGTGTGGGCACTCGACCGGAATTATCGACTAACTTTATTATTAAGAGCTGAAGGGGTATGTATCAATGTATCGGTTAAGCAGGGAGGAGTAAG

m501: strength = 1.076

GAGCTGCTGACAATTAGCCATCCGGCTCGTATAATGTGTGGAGTTGTGGGCGGATAACAGTTTCACACAGGGAGCAGCGCCGCCGAGAAAAAGCGAAGCGGCACTGCTCTTTAACAATTTATCAGGCAATCTGCGTGGGCACTCGACCGGAATTATCGATTGACTTTATTATTAGAAGCTGGAGAGGTGCGTATCAACGTATCGACTAAATAAGGAGGAATAAA

m505: strength = 2.469

GAGCTGTTGACAATTGATCATCCGGCTCGTATAATGTGTGGAATTGTGAGCGGATAACAACTTCACACAGGAAACAGCGCCGCCGAGAAGAAGCGGAGCGGCACTGCTCTCTAACGATTTGCCAGACAGCCTGTGTGGGCACTCGACCGGAGTCATCGATTAGCTCTATTATTGAAAACCAGAGAGGTATGTACTAATGTATCGACCAAGTAAGGAGGAGTAAG

m509: strength = 0.033

GAGCTGTTGACAACTAATCGTCCGGCTCGTATAGTATGTGGAACTGTGAGCGGATAACAACTTCACACAGGAAACAGCGCTGCTGAGAGAAAGCGAAGCGGCGCTGCTCTTTAACAATTTATCAGACAACCTGTGTGGGCACTCGACCGGAATTATCGATCAACTTTATTACCAAAAGTCAAAGAGGTGTGCACTGGCGTATCGGTTAGACAAGGAGGAGTAAA

m510: strength = 0.432

GAGCTGTTAACAATTAATCATTCGGCTCGTATAATGTGTGGAATCGTGAGCGGACAATAATTTCACGCGGGAAACAGCGCCGCTGAGGGAAAGCGAAGCGGCACTGCTCTTTAACAATTTATCAGACAGTCTGTGTGGGCACTTGACCGGAGTTACCGATCAACCTTATCACTAAGAATCAAAGAGGTATACACTAACGTATCGATTAAGCAAGGAGGAGTAAA

m514: strength = 0.174

GGGCTGTTGACAGTTAATCATCCGGCCCGTATAATGTGTGGAATCGTGAGCGGATAACAACTTCACGCAGGAAACAGCGCCGCTGAGAAAAAGCGAAGCGGCACTGCTCTTTAACAATTTATCAGACAACCTGTGTGGGCACTCGACCGGAATTACCGATTAACTTTACTATCAAAAACTAGAGAGGTATATATTGGTGCATCGATTAAGTAAGGAGGAATAAA

m517: strength = 0.238

AAGCCGTTGGCAGTTAGCCATCCGGCCCGTGTAATGTGTGGAACTGTGAGCGGATAACAATTTCACACAGGAAACAGCGCCGCTGAGAGAGAGCGAGGCGGCACTGCTCTCTAATGGTTTATCAGACAATCTGTGTGGGCACTCGACCGGAGTTATTGACTAACTTTATTGTTAAAAATTAAAGGGGCATATGCTAATGTACCGATTAAGTAAGGAGGAATGGA

m520: strength = 0.158

GAGCTGTCGACAATTAGTCACCCGGTTCGTATAATGTGTGGAATTGTGAGCGGATAACAATTTCACACAGGAAACAGCGCCGCTGAGAAGAAGCGGAGCGGCACTGCTCTCTAACAACTTATCAGACAGTCTGCGTGGGCGCTCGACCGGAATTATCGATCAACTTTACCATTAAGAATTAAAGAGGTATATGTCAATGTATCGATCAAGTAAGGAGGAATAAA

m521: strength = 0.734

GAGCTGTTGACAATTAGCCATCCGGCTCGTATAATGTGCGGGGTTGTGAGCGGATAACAGTTTCACACAGGAAACAGCGCCGCTGAGAAAGAGCGAAGCGGCACTGCTCCTCAACAATTTACCAGACAATCTGTGTGGGCACTCGACCGGAATTATCGATTAACTTTATTATTAAAAATTGAAGAGGTATACACCAATGTACCGGTTGAATAGGGAGGGATAAA

m524: strength = 0.396

GGGCCGTTGGCAGCTAATTACCCGGCTCGTATGGTGCGTGGAGTCGTGAGCGGATAGTAACCTCGCGCAGGAGGCGGCGCCGCCGAGGGGAAGCGGAGCGGCACTGCTCTTTAACAATCTATCAGACAATCTGTGTGGGCACTCGACCGGAGTTATCGACTAACTTTGTTACTGAGAATTAAAGAGGTATACACTAATGTACCGACTAAGTAAGGGGGAATAAA

m526: strength = 1.345

GAGCCGTTGATAATTAATCATCCGGCCCGTATAATGTATGGAGCTGTGAGCGAGTAACAATTTCACACAGGAAACAGCGCCGCTGGGAAAGAGCGAAGCGGCACTGCTCTTCAACAATTTATCAGACAACCTGTGTGGGCACTCGACCGGAATTATCGACTAACTCTGCTACTAAAGATTAAAGAGGTATATATTAATGTATCGATTAAGTAAGGAGGAATAAA

m534: strength = 0.154

GAGCTGCTGACAATTAATCACCCGGCTCGTATAATGTGTGGAATTGTGAGCGGATAACAATTTCACACAGGAAGCAGCGCCGCTGAGAAAAAGCGAAGCAGCACTGCTCTTTAGCAACTTGTCAGACAATCTGTGTGGGCACCCGACCGGAATCATCGACTAACTTCATTACCAAAAGTTAAAGAGGCATATATTAATGTATCGATTAAATAAGGAGGAATGAA

m542: strength = 0.093

GAGCTGTTGACAATTAATCATTCAGCTCGTGTGATGTGTGGAATTGTGAGTGGATAACAGTTTCACACAGGAAACAGCGCCGCTGAGAGGGGGTGAAGCGGCACTGCTCTTTGACAATTTATCAGACAGTCTGTGTGGGCACTCGACCAGGGTTATCGATTAACCTCATTATTAAGAATTAGAGAGATATCTATCGATGTATTGGTTAAATAAGGAGGAGCAGA

m545: strength = 0.094

GAGCTGTTGACAATTAATCATCCGGCTCGCATAATGTGTGGAATTGTGAGCGGATAACAATTTCACACAGGAAACAGCGCTGCTGAGAAAAAGCGAAGCGGCACTGCTCTTTAACAATTTATCAGACAATCTGTGTGGGCACTCGACCGGAATTATCGATTAACCTTATTGTTAGAGATTAAAGAGGTATGTGGTAATGTATCGATTGAATAGGGAGGAGTGAA

m546: strength = 1.224

GAGCCGTTGACAACTAATCATCCGGCTTGTATAATACGCGGAGCTGTGAGCGGATAACAATCTCATACAGGAAACAGCGCCGTCGAGAAAAGGCGAAGCGGCACTGCCCTTTAACAATTTATCAGACAATCCGTGTGGGCACTCGATCGGAATTATCGATCGACTTTATTATTGAAAATTAAAGAGGTACATGCTACTGTATCAATTAAATAAGGAGGAGTGAA

m548: strength = 0.145

GAGCTGCTGACAATTAGTCGTCCGGCTCGTATAATGTATGGGATTGTGAGCGGATAACAATTTCGCATAGGAAACAACGCCGCTGAGAAAAAGCGAAGCGGCACTGCTCTTTAACAATTTACCAGGCAATCTGTGTGGGCACTCGACCGGAATTATCGACTAACTTTACTACCAAAAATCAAAGAGGTCTACATTGATGCATCGATTAAATAAGGAGGAATAAA

m552: strength = 0.062

GAGCTGTCGACAATTAGTCATCCGGCTCGTATAATGTGTGGAATTGTGAGCGGATAACAATTTCACACAGGAGACAGCGCCGCTGAGAAAAAGCGAAGCGGCACTGCTCTTCAACAATTTATCAGACAATCTGTGTGGGCACCCGACTGGGATTATCGATTAATTTTGTTATTAAAAGCTAAAGAGGCATATATTAGTGCACCGACTAAATAAGGAGGGATAAG

m565: strength = 2.848

GAGCTGTTGACAATTAATCATCCGGCTCGCATAGTGTGTGGGACTGTGAGCGGACAGCAATTTCACACAGGAGACAACGCCGCTGAGAAAAGGCGAAACGGCGCTGCGCTTTAACAATTTATCAGACAATCTGTGTGGGCACTCGACCGGAATTATCGATTAACTTTATTACCAAGAACTAAAGAGGTACATATTAATGCATCGATTAGGTAAGGAGGAATAAA

m566: strength = 0.208

GAGCTGTTGACAATTAATCATCCGGCTCGTATAATGTGTGGAATTGTGAGCGGATAACAGTTTCGCACAGGAAACAGCGCCGCTGAGGAAAAGCGAAGCGGTACTGCTCTTCAACAGCTTATCAGACGATCCGTGCGGGCACTCGGCCGGAATTATCGATTAACTCTGTCATTAAAAATCAAAACGGTATATACTAATGTATCGACTAAGTGAGGAGGAATAGA

m573: strength = 0.791

GAGCTGTTGACAATTGATCGCCCGGCCCGTGTAATGCGTGGAATTGTGGGCGGACAACAATTTCACGCAGGAAACAGCGCCGCTGAGCAGAAGCGAAGCGGCATTGCTCTCTAACAACTTATCAGACAATCCGTGTGGGCACTCGACCGGAATTATCGATTGACTTTATCATTAAAGGTTAAAGAGGTAGATATTAATGTACCGATTAAATAAAGAGGAACAAA

m580: strength = 0.014

GAGCTGCTAACAATTGATCATCCGGCTCGTATAATGTGTGGAGCTGTGAGCGAATAACAATTCCACATAGGAAACAGCGCCGCTGAGAAAGAGCGAAGCGGCACTGCTCTTTAACAACTTATCAGACGACCTGTGTGGGCACTCGACCGGGACTATCGATTGACTTTATGGTCAAGAATTAAGGAGGTATATATCAATGTATCGATCAAACAAGGAGGAGTAAG

m585: strength = 0.105

GAGCTGTCGACAGCTAATCATCCGGCTCGTATAATGTGTGGAATTGTGAGCGGATAACAGTTTCACACAGGAAGCAGCGCCGCTGAGAAAAGGCGGAGCGGCACTGTTCTTTAGCGATTTATCAGATAATCCGTGTGGGCACTCGACCGGAACTATCGATCAACTTTATTGTTAAAAATCAAAGGGGCATCTATTGATGTATCGATTAAACAAGGGGGAATAGA

m586: strength = 0.106

GAGCTGTTGACAATCAATCATCCGGTTCGTATAATGTGCGGAATTGCGAGCGGATAACAATTTCACACAGGAAACAGCGCCGCTGAGGAGAAGTGAGACGGCGCTGCTCTTTAACAATTCATCAGACAGTCCGTATGGGCACTCGACTGGAATTATCGATCAACTTCACTATTAGAAATCAAAGAGGTGCACATTAGTGTACCGACTAAATAGGGAGGGATAAA

m587: strength = 0.606

GAGCTGTTGACAATTAATCATCCGGCTCGTATAATGTGTGGAGTTGTGAGCGGATAACAATTTCACACAGGAAACAGCGCCGCTGAGAAAAAGCGAAGCGGCACTGCTCCTTAGCAGTTCACCAGACGACCTGTGTGGGCGCTCGACCGGAGTTATCGATTAACTTGATTATTAAGAGTTAAAGGGGTATATATTAATGTATCGATTAAATAAGGAGGGATAAA

m590: strength = 0.076

GAGCTGTTGACAATTAATCGTCCGGCTCGTATAGTGTGTGGAATTGTGAGCGGATAACAATTTCACACAGGAAACAGCGCCGCTGAGAGAAAGCAAAGCGGCACTGCTCTTTCACAATTTATCAGACAGTCTATGTGGGCACTCGACCGGAATTATCGATTAACTTTATTACTAAAGATTAAAGAGGTATATATTAACGCGTCGGCTAGACGAGGGGGAACAAA

m591: strength = 0.090

GAGCTGTTGACAGCTAACCATCCGGCCCGTGTAGCGTGTGGAATTGTAAGCGGATGACAATTTCACACGGGAAACGGCGCCGCTAAGAAGAAGCGAAGCGGCACTGCTCTTTAACAATTTATCGGGCAATCCGTGTGGGCGCTCGACCGGAATTATCGATTAACTTCATTATTAGAAACTAGAGAGGTATATACTGATGTATCAATTAGATGAGGAGGAATAAA

m599: strength = 0.001

GAATTGTTGACAATTAATCACTCGGCTCGTATAGTACGTGGAATTGTGAGCGGATAACAGCTTCACACAGGGAACGGCGCCGCTGAGAAAGAGCGAAGCGGCACTGCTCTTTGACAATTTGTCAGACAATCTGTGTGGGCACTCGACTGGAATTATCGATTAACTTTGTTATTAAAAATTAAAGAGGTATGTATTGGTGCATTGGTTGAATAAGGAGGAATAAG

m606: strength = 0.018

GAGCTGCTGACCATTAGCCATCCGGCTCGTATAGTGTATGGAATTGTGAGCGGATAACGATTTCACACAGGAAACAGCGCCGCTGAGAAAAAGCGAGGCGGCACTGCTCTTTAACAATTTATCAGACAATCTGTGTGGGTACTCGACCGGAATTATCGGTTAACTTTACCATTAAAAATTAAAGAGGTACATATTGATGTATCGATTAAATAAGGAGGGATAAA

m626: strength = 0.640

GAGCTGTTGACAATTAATCCTCCGGCTCGTATAATGTGTGGAATTGTGAGCGGATAACAATTTCACACAGGAAACAGCGCCGCTGAGAAAAAGCGAAGCGGCACTGCTCTGTAACAATTTATCAGACAATCTGTGTGGGCACTCGACCGGAATTATCGATTAACTTTATTATTAACAATTAAAGAGGTATATATTAATGTATCGATTAAATAAGGAGGAATAAA

m629: strength = 0.395

GAGCTGTTGACAATTAATCATCCGGCTCGTATAATGTGTGGAATTGTGAGCGGATAACAATTTCACACAGGAAACAGCGCCGCTGAGCAACAGCGAAGCGGCACTGCCCTTTAACAATTTATCAGACAATCTGTGTGGGCACTCGACCGGAATTATCGATTAACTTTATGATTCAAAATTAAAGAGGTATATATTAAGGTATCGATTAAATAAGGAGGAATAAA

m640: strength = 0.366

GAGCTGTTGACAATTAATCATCCGGCTCGTATAAGGTGTGGAACTGTGAGCGGATAACCATTTCACACAGGAAACAGCGCCGCTGAGAAAAAGCGAAGCGGCACTGCTCTTTAACAATTTATCAGACAATCTGTGTGGGCACTCGACCGGAATTATCGATTAACTTTATGATTAAAAATTAAAGAGGTATATATTAATGTATCGATTAAATAAGGAGGAATAAA

m647: strength = 0.176

GAGCTGTTGACAATTAATCATCCGGCTCGTATAATGTGTGGAATTGTGAGCGGATAACAATTTCACACAGGAAACAGCGCCGCTGAGAAAAAGCGAAGCGGCACTGCTCTTTAACAATTTATCAGACAATCTGTGTGGGCACTCGACCGGAATTATCGATTCACTTTATTATTAAAAATTAAAGAGGTATATATTAATGTAGCGATTAACTAAGGAGGCATAAA

m659: strength = 0.910

GAGCTGTTGACAATTACTCATCCGGCTCGTATAATGTGTGGAATTGTGAGCGGATAACAATTTCACACAGGAAACAGCGCCGCTGAGAAAAAGCGAAGCGGCACTGCTCTTTAACAATTTATCAGACACTCTGTGTGGGCACTCGACCGGAATTATCGATTAACTTTATTATTCAAAATTAACGAGGTATATATTAATGTATCGATTAAATACGGAGGAATAAA

m664: strength = 0.106

GCGCTGTGGACAATTAATCATCCGGCTCGTATAATGTGTGGAATTGTGAGCGGATAACAATTTCACACAGGAAACAGCGCCGCTGAGAAAAAGCGAAGCGGCACTGCTCTTTAACCATTTATCAGACAATCTGTGTGGGCACTCGACCGGAATTATCGATTAACTTTATTATTAAAAATTAAAGAGGTATATATTAAGGTATCGATTAAATAAGGAGGAATAAA

m670: strength = 0.631

GAGCTGTGGACAATTAATCATCCGGCTCGTATAATGTGTGGAATTGTGAGCGGATAACAATTTCACACAGGAAACAGCGCCGCTGAGAAAAAGCGAAGCGGCACTGCTCTTTAACAATTTATCAGACAATCTGTGTGGGCACTCGACCGGAATTATCGATTAACTTTATTATTAAAAATTAAAGAGGTATATATTAATGTATCGATTAAATAAGGAGGAATAAA

m675: strength = 0.880

GAGCTGTTGACAATTAATCATCCGGCTCGTATAATGTGTGGAATTGTGAGCGGATAACAATTTCACACAGGACACAGCGCCGCTGAGAAACAGCGAAGCGGCACTGCTCTTTAACAATTTATCAGACAATCTGTGTGGGCACTCGACCGGAATTATCGATTAACTTTATTATTAAAAATTAAAGAGGTATATATTAATGTATCGATTCAATAAGGAGGAATAAA

m701: strength = 0.847

gagctgtggacaattaatcatccggctcgtataatgtgtggaattgtgagcggataacaagttcacacaggaaacagcgccgctgagacaaagcgaagcggcactgctctttaacaatttatcagacaatctgtgtgggcactcgaccggaattatcgattaactttattattaaaaattaaagaggtatatattaatgtatcgattaaataaggaggaataaa

m702: strength = 1.947

gagctgttgacaattaatcatccggctcgtataatgtgtggaattgtgagcggataacaatttcacacaggaaacagcgccgctgagaaaaagcgaagcggcactgcgctttaacaattgatcagacaatctgtgtgggcactcgaccggcattatcgattaactttattattaaaaattacagaggtatatatgaatgtatcgattaaatcaggaggaataaa

m705: strength = 0.987

gagctgttgacaattaatcatccggctcgtataatgtgtggaattgtgagcggataacaatttcacacaggaaacagcgccgcggagaaaaagcgaagcggcactgcgctttaacaatttatcagacaatctgtgtgggcactcgaccggaattatcgattaactttattattaacaattaaagaggtatatattaatgtatcgattaaataaggaggaataaa

m706: strength = 1.754

gagctgttgacaattaatcatccggctcgtataatgtgtggaattgtgagcggataacaatttcacacaggaaacagcgccgctgagaaaaagcgaagcggcactgctctttaacaatttatcagacaatctgtgtgggcactcgaccggaatgatcgattaactttattattaaaaattaaagaggtatatattaatgtatcgattagataaggaggaataaa

m708: strength = 2.722

gagccgttgacaattagtcacccggctcgtatgatgtgcggaattgtgagcggataacgatttcacacgggaaacagcgccgctgagaaagagcgaagtggcactgctcttcaacaatttgtcagacaacccgtgtgggcactcgaccggaattgtcgattaactttatcgttaaagattaaagaggcacatattaatgtgtcgattaaatgaggaggagtaga

m709: strength = 2.056

gagctgttgacaattaatcatccggctcgtataatgtgtggaattgtgagcggataacaatttcacacaggaaacagcgccgctgagaaaaagcgaagcggcactgctctttcacaatgtatcagacaatctgtgggggcactcgaccggaattatcgattaactgtattcttacaaattaaagaggtatctattaatgtatcgagtacataaggaggaataaa

m710: strength = 1.390

gagctgttgacaattaatcatccggctcgtataatgtgtggaattgtgagcggataacaatttcacacaggaaacagcgccgctgagaaaacgcgaagcggcactgctctttaacaatttatcagacaatctgtgtgggcactcgaccggaattagcgattaacgttattattaaaaattaaagaggtatatattaatgtatcgattaaataaggaggaataaa

**Sequences of designed Trc promoter/RBS**

s01: strength = 1.828

GAGCTATTGACAATTAATCACCCGGCTCGTATAATGTGCGGAATTGTGAGCGGATAACAACTTCACACAGGAAACAGCGCCGCTGGGGAGAAGCGAAGCGACACTGCTCCCTAACAATCTATCAGACAATCTGTGTGGGCACTCGACCGGAATTATCAATTAACTTTATTACTAAAGATCAAAGAGGCATATATCAATGTATCGGTTGAATAAGGAGGAATGAA

s02: strength = 1.699

gagctgttgacaattaatcatccggctcgtataatgtgtggaattgtgagcggataacaatttcacacaggaaacagcgccgctgagaaaaagcgaagcggcactgctctttaacaatttatcagacaatctgtgtgggcactcgaccggaattatcgattaacttgattattaaacattaaagaggtagatcttaatgtatcgattaaataaggaggaataaa

s03: strength = 1.730

GAGCTGTTGACAACTAGCCATCCGGCTCGTATAATGTGTGGAATCGTGAGCGGACAACAACTTCACACAGGGAACGGCGCCGCTGAGGAAAAGCGGAGCGGCATTGCTCTTTAACAATTTATCAGACAGTCCGTGCGGGCACTCGACCGGAGTTAGCGGCTAGCCTTGTTACTAAAAGTCAAAGAGGTGCACGTTAACGTGTCGATTAAACAAGGAGGAATAAA

s04: strength = 0.701

GAGCTGTTGGCAATTAATTATCCGACTCGTATAATGTGCGGGGTCGTGAGCGGATAACAATTCCACACAGGAGACAGCGCCGCTGAGAAAAAGCGAAGCGGCACTGCCCTCTAACAATTTACCAGACAATCCGTGTGGGCACCCGACCGGAATTATCGATTAACTTTATTATTAAAAATTAAAGAGGTATATATTCATGTATCAATTAGATAAGGAGGAATAAA

s05: strength = 0.999

gagctgttgacaattaatcagccggctcgtataatgtgtggaattgtgagcggataacaatttcacacaggaaacagcgccgctgagaaaaagcgaagcggcactgctctttaacaatttatcagaccatctgtgtgggcactcgaccggaattatcgattaactttattattaaaaattaaagaggtatatattaatgtatcgattaaataaggaggaataaa

s06: strength = 0.809

GAGCTGTTGACAGTTAGTCATCCGGCTCGTATAATGTGTGGAATTGTGAGCGGATAACAATTTCACACCGGAAACAGCGCCGCTGGGAAGAGGCGGGGCGGCACTGCTCTTTAACAATTTATCAGACAATCTGTGTGGGCACTCGGCCGGAGTCGTCGATTAGCTTTATTATTAGAAGTTAAAGAGGTATATATTAATGCATCGATTGAATAAGGAGGGATAAA

s07: strength = 0.570

GAATTGCTGACAATTAACCACCCGGCTTGTATAATGTGTGGAACTGTGAGTGGGTAGCGGTCCCACCCGGGAAACAGCGCCACTGAGAAGAAGCGGGGCGGCACTGCGCCCTAACGGTTTACCAGACAATCTGCGTGGGCACCCGGCCGGAGTCATCGATTAACTTTGTTACTAGAGATCAAGGAGGTATATATTAATGCGTCGGTTAAATAGGGAGGACTAGA

s08: strength = 0.657

GAACTGCTGACAATTACTCGTCCGACTCGTATAGTGTGCGGAACTGTGGGCGGGCAACGATTTCACACAGGAAACAGCGCCACTGAGGAGAGGCGAAGCGGCACTGCTCTCTAATAATTTATCAGACGATCTGTGTGGGCATTCGACCGGAGTTATCGATTAACTTCATTATCAAAGATTAAAGAGGTACATATTAATGCATCGATTAAATAGGGAGGGACAAA

s11: strength = 1.196

gagctgttgacaatgaatcatccggctcgtatcatgtgtggaattgtgagcggataacaatttcacacaggaaacagcgccgctgagaaacagcgaagcggcactgctctttaacaatttatcagacaatctgtgtgggcactcgaccggaattatcgagtaactttattattaaaaattaaagaggtctatattaatgtatcgattacataaggaggcataaa

s12: strength = 0.922

GAGCTATTGACAATTAATCATTCGGCTCGTGTGATGTGTGGGATGGTGAGCGGATACCAATTTCACACGGGAAGCAGCGCCGCTGAGAAAAAGCGAAGCGGCACTGCTCTTTAACAATTTATCAGACGACCTGTATGGGCACTCGACCGGGATTATCGATTAACCTTGTTATTAAAGATTAAAGAGGTGTATATTAATGTACCGATTAAATAAGGAGGAATAAG

s13: strength = 0.848

GAGCTGTTGACAATTAATCATCCGGCTCGTATAATGTGTGGAATTGTGAGCGGATAACAATTTCACACAGGAAACAGCGCCGCTGAGCAAAAGCGAAGCGGCACTGCTCTTTAACAATTTATCAGACAATCTGTGTGGGCACTCGACCGGAAGTATCGATTAACTGTATGATTAAAAATTAAAGAGGGATCTAGTAATGTATCGATTAAATAAGGAGGAATAAA

s14: strength = 0.560

GGGCTGTTGACAATTACTCATCCGGCTCATATAATGTGTGGGACTGTGAGCGGATAACAGCTTCACGCAGGAAACAGCGCCGCTGAGAAGAAACGGAGCGGCACCGCTCCTTAACAACTTATCAGACAACCCGCGTAGGCACTCGACCGGAATTACCGATTAACTTTACCATCAGAAATTAGAGAGGTACATACTGATGTATCGACTGAATAAGGAGGGATAAG

s15: strength = 0.640

GAGCTGTGGACAATTAATCATCCGGCTCGTATAATGTGTGGAATTGTGAGCGGATAACAATTTCACACAGGAAACAGCGCCGCTGAGAAAAAGCGAAGCGGCACTGCTCTGTAACAATTTATCAGACAATCTGTGTGGGCACTCGACCGGAATTATCGATTAACTTTATTATTAAAAATTAAAGAGGTATATATTAATGTATCGATTAACTAAGGAGGAATAAA

s21: strength = 2.501

GAGCTGTCGACAATTAATCATCCGGCTCGTATAATGCGTGGAATTGTGAGCGGATAATAATTTCACACAGGAAACAGCGCCGCCGAGAAAAAGCGAAGCGGCATTGCTCTTTAACGATTTATCAGACAATCTGTGCGGGCACTCGACCGGAATTATCGATTAACTCTATTATTAAAAATTAAAGAGGTATATATTAATGTATCGATTAAATAAGGAGGAACAAA

s22: strength = 0.705

GAGCTGTTGACAATTAATTATCCGGCGCGTGTAATGTGTGGAGCTGTGAGCGGATAATACTTTCACACAGGGAATAGCGCCGCCGAGAAAGAGTGAAGCGGCATTGCGCTTTAACGACTTATCAAACAATCTGTGCGGGCACCCGACCGGAATCATCGGTTAACGTTGTTGTCGAAAGTTAAAGCGGTATGTACTGATGTATCGACTAAATAGGGAGGAATAAA

s23: strength = 0.025

ACGCCGCCGACAAGTACTTATCCGGCTTGTGTAACGCGTGGAGCTGTGGGCGGATAACACTTTCCCCCGGGAAATAACGCTGTTGAAAAAGAGCGAAACAGCATTGCGCTTTAACAATTTATCAAACAATCTGTATGGGCACCCGACCAAGGTCGGCGATTAACGCCGCTGTCAGAAATTAAAGAGGCCTGCGCTGATGTGTCGGCTGAATGGGGAGAAATAAC
