# Supplementary material for: Quantitative Design of Regulatory Elements Based on High-Precision Strength Prediction Using Artificial Neural Network
Source: PLoS One. 2013 Apr 1;8(4):e60288. doi: 10.1371/journal.pone.0060288 (PMC3613377; doi:10.1371/journal.pone.0060288)
Supplement: Text S3 — Sequence alignment of Trc promoter & RBS elements. (PDF) [file pone.0060288.s003.pdf]

Sequence alignment

Section 1

```
           (1) 1       10        20        30        40            55
   m000:   (1) GAGCTGTTGACAATTAATCATCCGGCTCGTATAATGTGTGGAATTGTGAGCGGAT
   m001:   (1) GAGCTGTTGACAATTAATCACCCGGCTCGTATAATGTGTGGAATTGTGAGCGGAT
   m003:   (1) GAGCTGTTGACAATTAATCATCCGGCTCGTATAATGTGTGGAGTTGTGAGCGGAT
   m004:   (1) GAGCTGTTGACAATTGGTCATCCGGCTCGTATAATGTGTGGAACTGTGAGCGGAT
   m005:   (1) GAGCCGCTGGCAACTAGTCATCCGGCTCGTGTAATGTGTGGAATTGTGAGCGGGT
   m006:   (1) GGGCTGCCGGCAATTAATCACCCGACTTGTGTAGTGGGCGGAGTCGTGAGCGGAT
   m007:   (1) GGGCTGTTGACAATTAATTATCCGACTCGCGCAAGGCGTGGAATTGTGAGCGGAT
   m010:   (1) GAGTTGTTGACAGTTAATCATCCGGCCCGTACAGTGTGTGGGACTGTGAGCGGAC
   m014:   (1) AAGCTGTTGACAATTAATCACCCGGCTCGTATAGTGTGTGGAATTGTGAACGGAT
  m015b:   (1) GGGCTGTTGACAATTAACCATTCGGCTCGTGTAATGTGTGGAACTGTGAGCGGAT
   m017:   (1) GCGCTGTTGACAAGTGATCATCCGATTCGTATAATGTGTGGAATTGTGAGCGGAT
   m018:   (1) GAGCTGTTGGCAACTGATCATCCGGCTTGTATAATGTGTGGAGTGGTGAGCGGAT
   m019:   (1) GAACTGTTGACAATTAATCATCCGGCTCGCATAATGTGTGGGACCGTGAGCGGGT
   m021:   (1) AAGTTGTTGATAGTTAACCACCCGGCTCGTATAATGCGTGGAGTTGTGAGCGGAT
   m024:   (1) GGGCTGTTGACAGTTAACCATCCGGTTCGTATAATGCGTGGAGTTGTGAGCGGAT
   m026:   (1) GAGCTGTCGACAATTAACCGTCCGGCCCGTATAATGCGTGGAACTGTGGGCGGAT
   m028:   (1) GAGCTGCTGGCAATCAATCATCCGGCTCGTATAACGTGTGGAACTGTGAGTGGAT
   m029:   (1) GAGCTGTCGACAACTAGTCATCCGGCTCGTATAGTGTGTGGAATTGTGAGCGGAT
   m030:   (1) GAGCTGTTGACAACTAATCATCCGACCCGTATAACATGCGGAATTGTGAGCGGAC
   m031:   (1) GGGCTGTGGACAGTTGATCATCCGGCTCGTATAATGTGTGGAATTGTGAGCGGAT
   m054:   (1) GAGCTGTTGACAGTTAATCGTCCGGCTCGTGTAATGTGTGGAATTGTGAGCGGAT
   m085:   (1) GAGCTGTTGGCAATTAACCATCCGGCTCGTATAATGTGTGGAATTGTGAGCGGGT
   m092:   (1) GAGCTGTTGACGGTTAATTATCCGGCTCGTATAATGTGTGGAATTGTGAGCGGGT
   m150:   (1) AAGCTGTTGACGATTGATCACCCGGCTCGCATAATGTGTGGAGTTGTGAGCGGGT
   m198:   (1) GAGCTGCTGACAATTAATCACCCGGCTCGTATAATGCGCGGAACTGTGAGCGGAT
   m213:   (1) GAGCTGTTGACAATTAATCATCCGGCTCGTCTAATGTGGGGAATTGTGAGCGGAT
   m232:   (1) GAGCTGTTGACAATTAATCATCCGGCTCGTATAATGTGTGGAATTGTGAGCGGAT
   m244:   (1) GAGCTGTTGACAATTAATCATCCGGCTCGAATAATGTGTGGAATTGTGAGCGGAT
   m354:   (1) GAGCTGTTGACAATTAATCGTCCGGCTCGTATAGTGTGTGGAATTGTGAGCGGAT
   m360:   (1) GAGTTGTTGACAATTAATCATCCGGCTCGTATGATGCGTGGAGTTGTGAGCGGGT
   m363:   (1) GAGCTGTTGACAATTAATCATCCGGCCCGCGTAATATGTGGAATTGTGAGCGGAT
   m396:   (1) GAGCTGTTGACAATTAATCATCCGGCTCGTACAATGCGTGGAATTGTGAGCGGAT
   m412:   (1) GAGCTGTTGGCAATTAATCATCCGGCTCGTCTAATGCGTGGAATTGTGAGCGGAC
   m413:   (1) GAGCTGTTGACAGTTAATCATCCGGCTCGTATAATGTGTGGAATCGTGAGCGGAT
   m421:   (1) GAGCTGCTGACAATTAGTCATCCGGCTCGTATAATGTGTGGAATTGTGAGCGGAT
   m424:   (1) GAGCTGTTGACAGCTAATCATCCGACTCGTATAATGTGTGGAATCGTGAGCGGAT
   m427:   (1) GAGCTGTTGACAATTAATCATCCAGCTCGTACGATGTGTGGGATTGTGAGCGGAT
   m428:   (1) GAGCCGTTGACAGTTAATCATCCGGCTCGTGTAATGTGTGGAGTCGTGAGCGGAT
   m430:   (1) AAGCTGCCGATAACTAACCATCCGGCTCGTATAATGTGTGGAGTTGTGAACGGAT
   m434:   (1) GAGCTGCTGACAATTAATCATCCGGCTCGTATAATGCGTGGAACTGTGAGCGGAC
   m435:   (1) GAGCCGCTGATGATTAATCGTCCGACTCGTACAGTGTGTGGAATTGTGAACGGAT
   m441:   (1) GAGCTGTTGGCAGCTAGTCATCCGGCTTGTATAATGTGTGGAATTGTGAGCGGAT
   m442:   (1) GAGCTGTTGGCAATTAATCATCCGGCTCGTACAACGTGTGGAATTGCGGGCGGAT
   m444:   (1) GGGTTGTTGACGACTAATCATCCGGCTCGTGTAATATGTGGGATCGTGAGCGGAT
   m445:   (1) GGGCTGTTGACAATCAATCACCCAGTTCGTACGATGTGTGGAATTGTGAGCGGGT
   m447:   (1) GAGCTGTTGACAACTGATCACCCGGCTCGTATAATGCGTGGAGCTGCGAGCGGAT
   m449:   (1) GAGCTGTTGACAATTAATCATCCGGCTCGTATAGTGTGTGGAGTTGTGAGCGGAT
   m454:   (1) GAGCTGCTGGCAGTTGATCGTCCGGCTTGTATAATGTGTGGAGTTGTGAGCGGAT
   m459:   (1) GAGCTGTTGACAGTTAGTCATCCGGCTCGTATAATGTGTGGGGCTGCGAGCGGAC
   m460:   (1) GAGCTGTTGACAATTAATCATCCGGCTCGTATAGCGTGTGGAATTGTGAGCGGAT
   m463:   (1) AAGCTGTTGACAATTAACCATCCGGCTTGTATGATGTGCGGAATTGTGAGCGGAT
   m473:   (1) GAGCTGCTGACAATTAATCATCCGGCCCGTATAATGTGTGGGACCGTGGGCGGAT
   m477:   (1) GAGCTGCTGACTATTAACCACCCGGCTCGTATAATGTGTGGAATTGTGAGCGGAT
   m479:   (1) GAGCTGCTGACAATTAATCATCCGGCCCGTATAATGTGTGGGACCGTGGGCGGAT
   m483:   (1) GAGCTGTTGACAACTAACCATCCGGCTCGTATAACGTGTGGAATTGTGAGCGGAT
   m484:   (1) GAGCTATTGACAATTAATCATCCGGCTCGTATAATGTGTGGAATTGTGAGCGGAT
   m489:   (1) GGGCTGTTGACGATTAATCATCCGGCTCGTACAATGCGTGGAACCGTGAGCGGAT
```

Sequence alignment

m491:	(1)	GAGCTGTTGACAGTTAATCACCCGGCCCATATAATGTGTGGAATTGTGAGCGGAT
m501:	(1)	GAGCTGCTGACAATTAGCCATCCGGCTCGTATAATGTGTGGAGTTGTGGGCGGAT
m505:	(1)	GAGCTGTTGACAATTGATCATCCGGCTCGTATAATGTGTGGAATTGTGAGCGGAT
m509:	(1)	GAGCTGTTGACAACTAATCGTCCGGCTCGTATAGTATGTGGAACTGTGAGCGGAT
m510:	(1)	GAGCTGTTAACAATTAATCATTCGGCTCGTATAATGTGTGGAATCGTGAGCGGAC
m514:	(1)	GGGCTGTTGACAGTTAATCATCCGGCCCGTATAATGTGTGGAATCGTGAGCGGAT
m517:	(1)	AAGCCGTTGGCAGTTAGCCATCCGGCCCGTGTAATGTGTGGAACTGTGAGCGGAT
m520:	(1)	GAGCTGTCGACAATTAGTCACCCGGTTCGTATAATGTGTGGAATTGTGAGCGGAT
m521:	(1)	GAGCTGTTGACAATTAGCCATCCGGCTCGTATAATGTGCGGGGTTGTGAGCGGAT
m524:	(1)	GGGCCGTTGGCAGCTAATTACCCGGCTCGTATGGTGCGTGGAGTCGTGAGCGGAT
m526:	(1)	GAGCCGTTGATAATTAATCATCCGGCCCGTATAATGTATGGAGCTGTGAGCGAGT
m534:	(1)	GAGCTGCTGACAATTAATCACCCGGCTCGTATAATGTGTGGAATTGTGAGCGGAT
m542:	(1)	GAGCTGTTGACAATTAATCATTCAGCTCGTGTGATGTGTGGAATTGTGAGTGGAT
m546:	(1)	GAGCCGTTGACAACTAATCATCCGGCTTGTATAATACGCGGAGCTGTGAGCGGAT
m545:	(1)	GAGCTGTTGACAATTAATCATCCGGCTCGCATAATGTGTGGAATTGTGAGCGGAT
m548:	(1)	GAGCTGCTGACAATTAGTCGTCCGGCTCGTATAATGTATGGGATTGTGAGCGGAT
m552:	(1)	GAGCTGTCGACAATTAGTCATCCGGCTCGTATAATGTGTGGAATTGTGAGCGGAT
m565:	(1)	GAGCTGTTGACAATTAATCATCCGGCTCGCATAGTGTGTGGGACTGTGAGCGGAC
m566:	(1)	GAGCTGTTGACAATTAATCATCCGGCTCGTATAATGTGTGGAATTGTGAGCGGAT
m573:	(1)	GAGCTGTTGACAATTGATCGCCCGGCCCGTGTAATGCGTGGAATTGTGGGCGGAC
m580:	(1)	GAGCTGCTAACAATTGATCATCCGGCTCGTATAATGTGTGGAGCTGTGAGCGAAT
m585:	(1)	GAGCTGTCGACAGCTAATCATCCGGCTCGTATAATGTGTGGAATTGTGAGCGGAT
m586:	(1)	GAGCTGTTGACAATCAATCATCCGGTTCGTATAATGTGCGGAATTGCGAGCGGAT
m587:	(1)	GAGCTGTTGACAATTAATCATCCGGCTCGTATAATGTGTGGAGTTGTGAGCGGAT
m590:	(1)	GAGCTGTTGACAATTAATCGTCCGGCTCGTATAGTGTGTGGAATTGTGAGCGGAT
m591:	(1)	GAGCTGTTGACAGCTAACCATCCGGCCCGTGTAGCGTGTGGAATTGTAAGCGGAT
m599:	(1)	GAATTGTTGACAATTAATCACTCGGCTCGTATAGTACGTGGAATTGTGAGCGGAT
m606:	(1)	GAGCTGCTGACCATTAGCCATCCGGCTCGTATAGTGTATGGAATTGTGAGCGGAT
m626:	(1)	GAGCTGTTGACAATTAATCCTCCGGCTCGTATAATGTGTGGAATTGTGAGCGGAT
m629:	(1)	GAGCTGTTGACAATTAATCATCCGGCTCGTATAATGTGTGGAATTGTGAGCGGAT
m640:	(1)	GAGCTGTTGACAATTAATCATCCGGCTCGTATAAGGTGTGGAACTGTGAGCGGAT
m647:	(1)	GAGCTGTTGACAATTAATCATCCGGCTCGTATAATGTGTGGAATTGTGAGCGGAT
m659:	(1)	GAGCTGTTGACAATTACTCATCCGGCTCGTATAATGTGTGGAATTGTGAGCGGAT
m664:	(1)	GCGCTGTGGACAATTAATCATCCGGCTCGTATAATGTGTGGAATTGTGAGCGGAT
m670:	(1)	GAGCTGTGGACAATTAATCATCCGGCTCGTATAATGTGTGGAATTGTGAGCGGAT
m675:	(1)	GAGCTGTTGACAATTAATCATCCGGCTCGTATAATGTGTGGAATTGTGAGCGGAT
m701:	(1)	GAGCTGTGGACAATTAATCATCCGGCTCGTATAATGTGTGGAATTGTGAGCGGAT
m702:	(1)	GAGCTGTTGACAATTAATCATCCGGCTCGTATAATGTGTGGAATTGTGAGCGGAT
m705:	(1)	GAGCTGTTGACAATTAATCATCCGGCTCGTATAATGTGTGGAATTGTGAGCGGAT
m706:	(1)	GAGCTGTTGACAATTAATCATCCGGCTCGTATAATGTGTGGAATTGTGAGCGGAT
m708:	(1)	GAGCCGTTGACAATTAGTCACCCGGCTCGTATGATGTGCGGAATTGTGAGCGGAT
m709:	(1)	GAGCTGTTGACAATTAATCATCCGGCTCGTATAATGTGTGGAATTGTGAGCGGAT
m710:	(1)	GAGCTGTTGACAATTAATCATCCGGCTCGTATAATGTGTGGAATTGTGAGCGGAT

Sequence alignment

Section 2

	(56)	56 70 80 90 100 110
m000:	(56)	AACAATTTCACACAGGAAACAGCGCCGCTGAGAAAAAGCGAAGCGGCACTGCTCT
m001:	(56)	AACAACTTCACACAGGAAACAGCGCCGCTGAGAAAAAGCGAAGCGGCACTGCTTT
m003:	(56)	AACGACTTCCCACAGGAAACAGCACCGCTGAAGAAAGGCGAGGCGGCGCTGCCCT
m004:	(56)	AACGATCTCACACAGGAAGCAGCGCCGCTGGGGAAAGCGAAGCGGCACTGCTCT
m005:	(56)	AACAGTTTCACACAGGGAACGGCGCCGTTGAGGAAAAACGGAGCGGCACTGCTCT
m006:	(56)	AGCAACCTCACGTAGGAGACGGCATCGCTGAAAAGGGGCGAAGCGGCACTGCTCT
m007:	(56)	TACGGCCTCACACAGGAAACAGCGCCGCTGGGGAGGAGTGAGGCGGCACTACTCT
m010:	(56)	AGCAACTTTACACAGGAAACAGCGCCGCTGAGAGAAAGCGAAGCAGCACTGCTCC
m014:	(56)	CACAGTCTCACACAGGAGACAGCGCCACCGAGAGAAAGCGAAGCGGCACTGCTCT
m015b:	(56)	AACAGTTTCACACAGGAAACAGCACCGCTGAGGAAAAGCGAAGCGGCATTGCTCT
m017:	(56)	AACAATTTTACACAGGCAATAGTGCCGCTGAGAAAAAGCGAAGCGGCACTGTTCC
m018:	(56)	AATAACTCCGCACAGGAGACAGCGCCGCTGAGGAAGAGCGAGGCGGCACTGCCCC
m019:	(56)	AACAACTCCACACAGGGAACAGCGCCGCCGAGAAGGGGCGAAGCGGCACCGCTCT
m021:	(56)	AACAATCTCACACAGGGAACAGCGCCGCTGAGAAGGAACGAAGCAGCACCGCTCC
m024:	(56)	AACGATTTCACACAGGGAACGGCGCCGCCGAGAGAAAGCGGAGCGGTACTGCTCC
m026:	(56)	GACAATTCCGCACAGGAAACAGTGCCGCTGGGAAGAAGCGAGGCGGCACTGCCCT
m028:	(56)	AACAATTTCACACAGGAAACAGCGCCGCCGAGAAGAAGCGAGGCGGCGCTGCTCC
m029:	(56)	AGCAGTTTCACACGGGAAACAGCGCCACTAAGAAGAGGCGGGGCGGCACTGCTCC
m030:	(56)	AACAGTTTCACACGGGGGGTGGCGCCGTCGAGGAGAGATGCAGCGGCACTGCTCT
m031:	(56)	AACAGCTTCGCACAGGAAACAGCGCCGCTGAGGGAAAGCGAAACGGCACTGCCCC
m054:	(56)	AACAATCTCACACAGGAAACAGCGCCGCTGAGAAAAAGCGGAGCGGCACTGCTCC
m085:	(56)	AATAACTTCACACAGGAAACAGCGCCGCTGAGAAGAAGCGAAGCGGCACTGCTCT
m092:	(56)	AACAATTTCACACAGGGAACAGCGCCGCTGAGCGGAAGCGAAGCGGCACTGTTCT
m150:	(56)	AACAACTCCACACAGGGAACAGCGCCGCTGAGAAAAAGCGGAGCGGCACTGCTCT
m198:	(56)	AGCGGTTCCACACAGGAAACAGTGCCGCTGAGAAAAAGCGAAGCGGCGCTGCTCT
m213:	(56)	AACAATTTCACACAGGAAACAGCGCCGCTGAGAACAAGCGAAGCGGCACTGCTCT
m232:	(56)	AACAATTTCACACAGGAAACAGCGCCGCTGAGAAAAAGCGAAGCGGCACTGCTCT
m244:	(56)	ACCAATTTCACACAGGAAACAGCGCCGCTGAGAAAAAGCGAAGCGGCACTGCTCT
m354:	(56)	AACAACTTCACGCAGGAAACAGCGCCGCTGAGAAAGAGCGAGGCGGCGCTGCCCT
m360:	(56)	AACAACTCCACACGGGAAACAGCGCCGCTGAGAGAAAGCGAAACGACACTGTTCT
m363:	(56)	AACAGTTCCACACAGGAAACAGCGCCGTTGAGAAGAAGCGAAGCGGCACTGCCCT
m396:	(56)	AACAATTTCACACAGGAAACAGCGCTGCTGAGAAAAAGCGAAACGGCACTGCTCT
m412:	(56)	AACAATTTCACACAGGAAACAGCGCCGCTGAGAAAAGGCGAAGCGGCACTGCTCT
m413:	(56)	AACAGTTTCACACAGGAAACAGTGCCGCTGAGAAAAGGCGAAGCGGCGCTGCCCT
m421:	(56)	AACAGTTTCACACAGGAGGCAGCGCCGCTGAGAAAAAGCGAGGCGGCACTGCTCT
m424:	(56)	AACAATTTCACACAGGAAACAGCGCCGCTGAGAAAAAGCGAAGCGGCACTGCCCT
m427:	(56)	AACAATTCCACACAGGAAACGGCGCCGCTGAGAAAAAGTGGAGTGGCGCTGCTCT
m428:	(56)	AACAATTTCACACAGGAAACAATGCCGCTGAGAAAAAGCGAAGCGGCACTGCTCT
m430:	(56)	AACAATTTCACACAGAGAACAACGCCGCGGAGAAGGAGCAAAGCGGCACTGCTCT
m434:	(56)	AACAATTTCACACAGGAGACAACGCCGCTGAGAGAAAGCGGAGCGGCACTGCTCC
m435:	(56)	AACAATTTCGCACAGGAAACAGCGCCACTGAGAAAAAGCAAGGCGGCACTGCTCT
m441:	(56)	AACAATTCCACACAGGAAGCAGCGCCGCTGAGAGAGAGCGAAGCGGCACTGCTCC
m442:	(56)	AACAGTTTCACACGGGAAACAGCGCCGCTGAGAAAGAGCGAAGCGGCGCTGCTCT
m444:	(56)	GACAATCTCGCACAGGAAGCAACGCCGCCGAGAAGAAGCGGAGCGGCACTGCTCC
m445:	(56)	GACAATTTCACACGGGAAACAGCGCCGCTGAGGAAAAGCGAAGCGGCACTGCTCT
m447:	(56)	AACAATTTCACACAGGAGACAGCGCCACTGAGAAAAAACGAAGCGGCACTGCTCT
m449:	(56)	GACAGTCTCGCACAGGAAACAGCGCCGCTGGGAAAAAGCGAAGCGGCACTGCTCT
m454:	(56)	AACAATCTCACGCAGGGAACAGCGCCGCTGAGGAGAAGCGAAGCAACACTGCTCT
m459:	(56)	AACAGTTTCGCACAGGAAACAGCGCCGCTGAGAGAAAGCGAAGCAGCACTGCTCT
m460:	(56)	AATAATTCCACACAGAAAACCGCGCCGCTGAGGAAAAACGAAGTGGCGCTGCTCT
m463:	(56)	AACAATTCCACACAGGAGATAGCGCCGCTGAGGAAAAGCGGAGCGGCACTGCCCT
m473:	(56)	AGCAGTTTTACACAGGAAACAACGCCGTTAAGAAAGAGCGAAGCGGCGCTGCGCT
m477:	(56)	AACAATTTCACACAGGAAACAGCGCCGCTGAGAAAAAGCGAAGCGGCACTGCTCT
m479:	(56)	AGCAGTTTTACACAGGAAACAACGCCGTTAAGAAAGAGCGAAGCGGCGCTGCGCT
m483:	(56)	AACAACTTCACGCAGGGGATAGCGCCGCTGAGAAAAGGCGAAGCGGCACTGCTCT
m484:	(56)	AACAATTTCGCACAGGAAACAGCGCCGCTGAGAAAAAGCGAAGCGGCACTGCTCC
m489:	(56)	AACAACTTCACACAGGAAGCAGCGCCGCTGAGGAAAAGCGAAGCAGCATTGCTCT

Sequence alignment

```
m491:  (56) GACAACTTCACACGGGGAACAGCGTCGCTGAGGAAGAGCGAGGTGGCACTGCTCT
m501:  (56) AACAGTTTCACACAGGGAGCAGCGCCGCCGAGAAAAAGCGAAGCGGCACTGCTCT
m505:  (56) AACAACTTCACACAGGAAACAGCGCCGCCGAGAAGAAGCGGAGCGGCACTGCTCT
m509:  (56) AACAACTTCACACAGGAAACAGCGCTGCTGAGAGAAAGCGAAGCGGCGCTGCTCT
m510:  (56) AATAATTTCACGCGGGAAACAGCGCCGCTGAGGGAAAGCGAAGCGGCACTGCTCT
m514:  (56) AACAACTTCACGCAGGAAACAGCGCCGCTGAGAAAAAGCGAAGCGGCACTGCTCT
m517:  (56) AACAATTTCACACAGGAAACAGCGCCGCTGAGAGAGAGCGAGGCGGCACTGCTCT
m520:  (56) AACAATTTCACACAGGAAACAGCGCCGCTGAGAAGAAGCGGAGCGGCACTGCTCT
m521:  (56) AACAGTTTCACACAGGAAACAGCGCCGCTGAGAAAGAGCGAAGCGGCACTGCTCC
m524:  (56) AGTAACCTCGCGCAGGAGGCGGCGCCGCCGAGGGGAAGCGGAGCGGCACTGCTCT
m526:  (56) AACAATTTCACACAGGAAACAGCGCCGCTGGGAAAGAGCGAAGCGGCACTGCTCT
m534:  (56) AACAATTTCACACAGGAAGCAGCGCCGCTGAGAAAAAGCGAAGCAGCACTGCTCT
m542:  (56) AACAGTTTCACACAGGAAACAGCGCCGCTGAGAGGGGGTGAAGCGGCACTGCTCT
m546:  (56) AACAATCTCATACAGGAAACAGCGCCGTCGAGAAAAGGCGAAGCGGCACTGCCCT
m545:  (56) AACAATTTCACACAGGAAACAGCGCTGCTGAGAAAAAGCGAAGCGGCACTGCTCT
m548:  (56) AACAATTTCGCATAGGAAACAACGCCGCTGAGAAAAAGCGAAGCGGCACTGCTCT
m552:  (56) AACAATTTCACACAGGAGACAGCGCCGCTGAGAAAAAGCGAAGCGGCACTGCTCT
m565:  (56) AGCAATTTCACACAGGAGACAACGCCGCTGAGAAAAGGCGAAACGGCGCTGCGCT
m566:  (56) AACAGTTTCGCACAGGAAACAGCGCCGCTGAGGAAAAGCGAAGCGGTACTGCTCT
m573:  (56) AACAATTTCACGCAGGAAACAGCGCCGCTGAGCAGAAGCGAAGCGGCATTGCTCT
m580:  (56) AACAATTCCACATAGGAAACAGCGCCGCTGAGAAAGAGCGAAGCGGCACTGCTCT
m585:  (56) AACAGTTTCACACAGGAAGCAGCGCCGCTGAGAAAAGGCGGAGCGGCACTGTTCT
m586:  (56) AACAATTTCACACAGGAAACAGCGCCGCTGAGGAGAAGTGAGACGGCGCTGCTCT
m587:  (56) AACAATTTCACACAGGAAACAGCGCCGCTGAGAAAAAGCGAAGCGGCACTGCTCC
m590:  (56) AACAATTTCACACAGGAAACAGCGCCGCTGAGAGAAAGCAAAGCGGCACTGCTCT
m591:  (56) GACAATTTCACACGGGAAACGGCGCCGCTAAGAAGAAGCGAAGCGGCACTGCTCT
m599:  (56) AACAGCTTCACACAGGGAACGGCGCCGCTGAGAAAGAGCGAAGCGGCACTGCTCT
m606:  (56) AACGATTTCACACAGGAAACAGCGCCGCTGAGAAAAAGCGAGGCGGCACTGCTCT
m626:  (56) AACAATTTCACACAGGAAACAGCGCCGCTGAGAAAAAGCGAAGCGGCACTGCTCT
m629:  (56) AACAATTTCACACAGGAAACAGCGCCGCTGAGCAACAGCGAAGCGGCACTGCCCT
m640:  (56) AACCATTTCACACAGGAAACAGCGCCGCTGAGAAAAAGCGAAGCGGCACTGCTCT
m647:  (56) AACAATTTCACACAGGAAACAGCGCCGCTGAGAAAAAGCGAAGCGGCACTGCTCT
m659:  (56) AACAATTTCACACAGGAAACAGCGCCGCTGAGAAAAAGCGAAGCGGCACTGCTCT
m664:  (56) AACAATTTCACACAGGAAACAGCGCCGCTGAGAAAAAGCGAAGCGGCACTGCTCT
m670:  (56) AACAATTTCACACAGGAAACAGCGCCGCTGAGAAAAAGCGAAGCGGCACTGCTCT
m675:  (56) AACAATTTCACACAGGACACAGCGCCGCTGAGAAACAGCGAAGCGGCACTGCTCT
m701:  (56) AACAAGTTCACACAGGAAACAGCGCCGCTGAGACAAAGCGAAGCGGCACTGCTCT
m702:  (56) AACAATTTCACACAGGAAACAGCGCCGCTGAGAAAAAGCGAAGCGGCACTGCGCT
m705:  (56) AACAATTTCACACAGGAAACAGCGCCGCGGAGAAAAAGCGAAGCGGCACTGCGCT
m706:  (56) AACAATTTCACACAGGAAACAGCGCCGCTGAGAAAAAGCGAAGCGGCACTGCTCT
m708:  (56) AACGATTTCACACGGGAAACAGCGCCGCTGAGAAAGAGCGAAGTGGCACTGCTCT
m709:  (56) AACAATTTCACACAGGAAACAGCGCCGCTGAGAAAAAGCGAAGCGGCACTGCTCT
m710:  (56) AACAATTTCACACAGGAAACAGCGCCGCTGAGAAAACGCGAAGCGGCACTGCTCT
```

Sequence alignment

Section 3

```
        (111) 111      120       130       140       150           165
  m000: (111) TTAACAATTTATCAGACAATCTGTGTGGGCACTCGACCGGAATTATCGATTAACT
  m001: (111) TTAACAATTTATCAGACAATCTGTGTGGGCACTCGACCGGAATTACCGATTAACT
  m003: (111) TTAACGACTTACCAAACAGTCTGTACGGGCACTCGACCGGAATTATCGATTAACT
  m004: (111) TTAACGATTTATCAAACGACCGGTGTGGGCACTCGGCCGGAATTACCGATTAGCT
  m005: (111) TTAACAACTTATCAGACAATCCGTGTGGGCACTCGACCGGAATTATCGGTTAACT
  m006: (111) TTAACAGCTCATTAGGCAACCTGCGTAAACACTCGACCGGGGTTATCGACTAACC
  m007: (111) TTAACAATTTATCAGGCAATCCGTGTAGGCACCCGATCGGAATTATCGACCAACC
  m010: (111) TTAACAATTTATCAGACAGTTTGTGTGGGCACTCGACTAGAATTATCGATTAACT
  m014: (111) TTAACAACTTATCAGACAAGCTGTGTGGGCACCCGACCGAAATTATCGACTAACT
 m015b: (111) TTAGCAATTTATCAGACAATCTGCGTGGGCACTCGACCGGAATTATCGGTTAACT
  m017: (111) TCAACAACTTATCAGACAGCCCGTGTGGGCACTCGATCGGAGTTATCGGTCAACT
  m018: (111) TTAACGATCTATCAGACAATCTGCGTGGGCACTCGACCGGGATTGCCGACTAACT
  m019: (111) TTAACAGTTTATCAGACAATTTGTGTGGGTACTCAACCGGAATTACCAATTAACC
  m021: (111) CTAACAGTTCATCAGGCGACCTGCGTGGGCGCCCGACCGAGACTGTCGGTTAACC
  m024: (111) TCAACAATTCATCAGACGATCTGTGTGGGCACTCGACTGGAGTTATCGATTAGCT
  m026: (111) TTGACAGTCTATCAGACAATCTGCGTGGGCACTCGACCGGAATTATCGATTAACC
  m028: (111) CTGACAATTTATCAGACAATCTGTGTGGGCACTCGATCGGAGTTATCGATTAACT
  m029: (111) TTAGCAACTTATCAGGCAGTTTGTGTGGGCGCCCGACTGGACTATCGGTTGGCC
  m030: (111) TTAGCAGCCTATTAGGCAGTCTGTATGGGCACCCGACCGGGATTACCGATTAGCC
  m031: (111) TTAGCAATCTATCAGACAATCTGCGTGGACACCCGACCGGAGCTATCAGTTAACT
  m054: (111) TTAACAATTTATCAGACAGTCTGTGTGGGCACTCGACCGGAATCATCGATTAACT
  m085: (111) TTAGCAGCTTATCAGACAATCTGTGTGGGCACTCGGCCGGAGTTATCGATTAACT
  m092: (111) TTGACAATCTGTCAGACGATCTGCGTGGGCACTTGACCGGAATTATCGACTAGCT
  m150: (111) TTGACGATCCATCGGGCAGCCCGTGTGGGCACTCGATCGGAGTTACCGATTAACT
  m198: (111) TTAACAATTTATCGGGCAATCTGTGTGGGCACTCGACCGGAATTACCGATCAACT
  m213: (111) TTCACAATTTATCAGACAATCTGTGTGGGCACTCGACCGGAATTATCGATTAACT
  m232: (111) TTAGCAATTTATCAGACAATCTGTGTGGGCACTCGACCGGAATTATCGATTAACT
  m244: (111) TTAACAATTGATCAGACAATCTGTGTGGGCACTCGACCGGAATTATCGATTAACT
  m354: (111) TTGACAATTTGCCAGACAATCTGCGTGGGCACTCGACCGGAATTATCGATTAACT
  m360: (111) TTAACAGTTCATCAGACAATCTGCGTGGGCACTCGACCGGGACTATCGATTAACT
  m363: (111) TTAGCAATTTATCAGACAATCTGTGTGGGCACCCGACCGGAATTATCGAGTAACT
  m396: (111) TCAACAGTTTATCAGACAATCTGTGTGGGCACTCGACCGGAATTATCGATTAACT
  m412: (111) TTAACAATTTGTTAGACAATCTGCGTGGGCACTCGACCGAAATTGTCGATTAACT
  m413: (111) TTAACAATTTATCAGACAATCTGTGTGGGCACTCGACCGGAGTCATCGATTAACG
  m421: (111) TTCACAATTTATCAGACAATCTGTGTGGGCACTCGACCGGAGTTATCGACTAACC
  m424: (111) TTAACAATTTATCAGACAATCTGTGCGGGCACTCGACCGGAACTATCGATTGACT
  m427: (111) TTAACAATTCATCAGACAACCTGTGTGGGCACCCGACCGGAATTGTCGGTTAGTT
  m428: (111) TTAACAATCTATTAGACAGTCCGTGTGGGCACTCGACCGGAATTATCGACTGACT
  m430: (111) TTAACAACTTGTCAGACAGTCTGGGTGGGCACTCGACCGGAGTTGTCGATTAGCT
  m434: (111) TTGACAATTTATCGGACAATCTGTGTGGGCACTCGACCGGAATTATCGATTAACT
  m435: (111) TTAACAATTTATCAGACAATCTGTGTGGGCGCTCGACCGGAACTATCGATTAGCT
  m441: (111) CTAACAGTCTGTCAGATAATCTGCGTGGGCACCCGACCGGAGCCATCGATTAACT
  m442: (111) TTAACAATTTATCAGACAGTCTGCGTGGGCACTCGACCGGAGTCATCGATTAACT
  m444: (111) TTAACGACTTATCAGACAATCTGTGTGGGCACTCGACCGGAGTCATCGATCAGCT
  m445: (111) TTAACAATCTATCAGACAATCTGTGTGGGCACTCGACCGGGGTCATCGATTAACT
  m447: (111) CTAGCAGTCTATCAGGCAATCTGTGTGGACGCTCGACCGGGATTATCGATTAACT
  m449: (111) TTAACAATTTATCAGACGATCTGTGTGGGCGCCCGACCGGAATCATCGGTTAACT
  m454: (111) TTAACGATTTATCAGACAATCTGTGTGGGCGCTCGGCCGGAATTATCGATCAACT
  m459: (111) CTAACAGTTTATCAGACAATCTGTGTGGGCACTCGACCGGGGTTATCGGTTAACT
  m460: (111) TTAGCAATTTATCGGACAACCTGTGTGGGCACTCGACCGGGATTATCAATTAGCT
  m463: (111) TTAGCAATTTGTCAGACAACCTGTGTGGGCACTCGACCGGAGTTGTCGATTAACT
  m473: (111) TTAACAACTTGTCGGACAATCCGTGTAGACACTCGACCGGAGTTATCGATTAACC
  m477: (111) TTAACAACTTATCAGACAATCTACGTGGGCACTCGACCGGAGTTATCGATTAACT
  m479: (111) TTAACAACTTGTCGGACAATCCGTGTAGACACTCGACCGGAGTTATCGATTAACC
  m483: (111) TTAACAATTTATCAGACAATCTGTGCGGGCACTCGACCGGAGTTATCGATTAACT
  m484: (111) TTAACAATTTACCAGACAATCCGTGTGGGCACTCGACCGGAATTATCGGTTAACT
  m489: (111) TTAGCAACTCATCAGACAATCTGTATGGACACTCGACCGGGATTATCGATTGGCC
```

Sequence alignment

```
m491: (111) TTAGCAATTTATCAGACAGTCTGTGTGGGCACTCGACCGGAATTATCGACTAACT
m501: (111) TTAACAATTTATCAGGCAATCTGCGTGGGCACTCGACCGGAATTATCGATTGACT
m505: (111) CTAACGATTTGCCAGACAGCCTGTGTGGGCACTCGACCGGAGTCATCGATTAGCT
m509: (111) TTAACAATTTATCAGACAACCTGTGTGGGCACTCGACCGGAATTATCGATCAACT
m510: (111) TTAACAATTTATCAGACAGTCTGTGTGGGCACTTGACCGGAGTTACCGATCAACC
m514: (111) TTAACAATTTATCAGACAACCTGTGTGGGCACTCGACCGGAATTACCGATTAACT
m517: (111) CTAATGGTTTATCAGACAATCTGTGTGGGCACTCGACCGGAGTTATTGACTAACT
m520: (111) CTAACAACTTATCAGACAGTCTGCGTGGGCGCTCGACCGGAATTATCGATCAACT
m521: (111) TCAACAATTTACCAGACAATCTGTGTGGGCACTCGACCGGAATTATCGATTAACT
m524: (111) TTAACAATCTATCAGACAATCTGTGTGGGCACTCGACCGGAGTTATCGACTAACT
m526: (111) TCAACAATTTATCAGACAACCTGTGTGGGCACTCGACCGGAATTATCGACTAACT
m534: (111) TTAGCAACTTGTCAGACAATCTGTGTGGGCACCCGACCGGAATCATCGACTAACT
m542: (111) TTGACAATTTATCAGACAGTCTGTGTGGGCACTCGACCAGGGTTATCGATTAACC
m546: (111) TTAACAATTTATCAGACAATCCGTGTGGGCACTCGATCGGAATTATCGATCGACT
m545: (111) TTAACAATTTATCAGACAATCTGTGTGGGCACTCGACCGGAATTATCGATTAACC
m548: (111) TTAACAATTTACCAGGCAATCTGTGTGGGCACTCGACCGGAATTATCGACTAACT
m552: (111) TCAACAATTTATCAGACAATCTGTGTGGGCACCCGACTGGGATTATCGATTAATT
m565: (111) TTAACAATTTATCAGACAATCTGTGTGGGCACTCGACCGGAATTATCGATTAACT
m566: (111) TCAACAGCTTATCAGACGATCCGTGCGGGCACTCGGCCGGAATTATCGATTAACT
m573: (111) CTAACAACTTATCAGACAATCCGTGTGGGCACTCGACCGGAATTATCGATTGACT
m580: (111) TTAACAACTTATCAGACGACCTGTGTGGGCACTCGACCGGGACTATCGATTGACT
m585: (111) TTAGCGATTTATCAGATAATCCGTGTGGGCACTCGACCGGAACTATCGATCAACT
m586: (111) TTAACAATTCATCAGACAGTCCGTATGGGCACTCGACTGGAATTATCGATCAACT
m587: (111) TTAGCAGTTCACCAGACGACCTGTGTGGGCGCTCGACCGGAGTTATCGATTAACT
m590: (111) TTCACAATTTATCAGACAGTCTATGTGGGCACTCGACCGGAATTATCGATTAACT
m591: (111) TTAACAATTTATCGGGCAATCCGTGTGGGCGCTCGACCGGAATTATCGATTAACT
m599: (111) TTGACAATTGTCAGACAATCTGTGTGGGCACTCGACTGGAATTATCGATTAACT
m606: (111) TTAACAATTTATCAGACAATCTGTGTGGGTACTCGACCGGAATTATCGGTTAACT
m626: (111) GTAACAATTTATCAGACAATCTGTGTGGGCACTCGACCGGAATTATCGATTAACT
m629: (111) TTAACAATTTATCAGACAATCTGTGTGGGCACTCGACCGGAATTATCGATTAACT
m640: (111) TTAACAATTTATCAGACAATCTGTGTGGGCACTCGACCGGAATTATCGATTAACT
m647: (111) TTAACAATTTATCAGACAATCTGTGTGGGCACTCGACCGGAATTATCGATTCACT
m659: (111) TTAACAATTTATCAGACACTCTGTGTGGGCACTCGACCGGAATTATCGATTAACT
m664: (111) TTAACCATTTATCAGACAATCTGTGTGGGCACTCGACCGGAATTATCGATTAACT
m670: (111) TTAACAATTTATCAGACAATCTGTGTGGGCACTCGACCGGAATTATCGATTAACT
m675: (111) TTAACAATTTATCAGACAATCTGTGTGGGCACTCGACCGGAATTATCGATTAACT
m701: (111) TTAACAATTTATCAGACAATCTGTGTGGGCACTCGACCGGAATTATCGATTAACT
m702: (111) TTAACAATTGATCAGACAATCTGTGTGGGCACTCGACCGGCATTATCGATTAACT
m705: (111) TTAACAATTTATCAGACAATCTGTGTGGGCACTCGACCGGAATTATCGATTAACT
m706: (111) TTAACAATTTATCAGACAATCTGTGTGGGCACTCGACCGGAATGATCGATTAACT
m708: (111) TCAACAATTGTCAGACAACCCGTGTGGGCACTCGACCGGAATTGTCGATTAACT
m709: (111) TTCACAATGTATCAGACAATCTGTGGGGGCACTCGACCGGAATTATCGATTAACT
m710: (111) TTAACAATTTATCAGACAATCTGTGTGGGCACTCGACCGGAATTAGCGATTAACG
```

Section 4

	(166)	166 180 190 200 210 220
m000:	(166)	TTATTATTAAAAATTAAAGAGGTATATATTAATGTATCGATTAAATAAGGAGGAA
m001:	(166)	TTATTATTAAAAATTAAAGAGGTATATATTAATGTATCGATTAAATAAGGAGGAA
m003:	(166)	CTACTGTCAAAAGTTAAGGGGGTATATATTGATGTATCGACTAAGCAAGGAGGGG
m004:	(166)	CTATTACCAAAAATTAAAGAGGTATGTATTAATGTACCGACTAAGTAAGGAGGAA
m005:	(166)	TTACTATCAAAAATTAGGGAGGTATATATTAGTGTATCGATTGAATAAGGGGGAA
m006:	(166)	CTATTGCCAGGGACTGAAGAGATGCGCACTAGTGTATCGGTTAAGTAAGGAGGAA
m007:	(166)	CCATTACTAAAGATTAAGGGGGTATATACTAATGTATCGATTAAGTAGGGAGGAA
m010:	(166)	TTATTATTAAAAATTAAAGAGGTATATATTAATGTATCGATTAAATAAGGAGGAA
m014:	(166)	CCATTATTAAAAATTAAGGGGGTATATACTAAGGTACCGATTAAATGAGGGGGAA
m015b:	(166)	TTACTATTAGGGACTAAAGAGGTATGTATTAGTGTATCGATTAAATAAGGAGGAA
m017:	(166)	CTATCATTAAAGACCAAAGGGGTATATATTAATGTATCGACTAAGCAAGGGGGGC
m018:	(166)	TTATTACTAGAAGTTAAAGAGGTGTATACTAATGTATCGGTTGAATGAGGGGGAA
m019:	(166)	TCGTTACTGAGGATTAAAGGGGTGTAGATCAGTGTATCGATTAAATAAGGAGGAA
m021:	(166)	CTACTATCAAGGACCAAAGGGGTATATATTGGTACATCGACTGAGTAAGGAGGAG
m024:	(166)	CTACTATTAAGGATTAGCGGGGTATATGCTAATGTGTCGATTAAATAAGGAGGGA
m026:	(166)	TCACTATTAGAAATTGGAGGGACATATATCACTGTACCGACTAAACAAGGAGGAA
m028:	(166)	CTACTATTGAAAGTCGAGGGGGTATACATTAATGCATCGGTTAGATAAGGGGGAA
m029:	(166)	CTGCTATTCAAGGCTAAAGGGACGCGTACCAATGCATCGGTTGAATAAGGGGGAA
m030:	(166)	TCACTATTAAGAGCTAAAGGGGTGTGTACTAACGTACCGATCAAATAAGAAGGAA
m031:	(166)	CTATTATCAGAAATTAGAGAGGTATACATTCATGTATCGATTGAATAAGGAGGAA
m054:	(166)	CTATTATTAGAAATTAAAGAGGTATATATCAATGTATCGATTAAGTAAGGAGGAA
m085:	(166)	TTACTATTAACAGTTAAAGAGGTATATATTAATGTATCGATTAAATAAGGAGGAA
m092:	(166)	TTGTTGTTAGAAATTAAAGAGGCATATATTAATGTACCGATTAAATAAGGAGGAA
m150:	(166)	TTATTATTAAAAATTAAAGAGGTATATATTAATGTATCGATTAAATAAGGAGGAA
m198:	(166)	TTGTCATCAAGAGTTAAGGGGGTGCATATTGATGTATCGATTAAACAAGGGGGGA
m213:	(166)	TTATTATTCAAAATTAACGAGGTATATATTAATGTATCGATTAAATAAGGAGGAA
m232:	(166)	TTATTATTAAAAATTAAAGAGGTATATATTAATGTATCGATTAAATCAGGAGGAA
m244:	(166)	TTATTCTTAAAAATTAAAGAGGTATATATGAATGTATCGATTAAATAAGGAGGAA
m354:	(166)	TTACTATTAAAAATTAAAGAGGTATATACTAATGTACCGACTAGACAAGGAGGAG
m360:	(166)	TTATTGTTAAAAGTTAAGGAGGTATATATTGATGTATCGATCAAATAAGGAGGAA
m363:	(166)	TTGTTACTAAAAATTAAAGGGGTAGACATTAATGTATCGATCAAATGAGGGGGAG
m396:	(166)	TTATTATTAAAAACTAAAGAGGTATATACTAATGTACCGGTTAAACAAGGAGGAA
m412:	(166)	TTATTATTAAAAACTAAAGAGGTATATATTAGTGTATCGATTAAATAGGAAGGAG
m413:	(166)	TTATTATCAGAAATTAAAGAGGTATATGTCAGTGTATCGACTAAATAAGGAGGAA
m421:	(166)	TTATTAGTAGAGATTGAAGAGGTGTATATTAATGTATCGGTTAAATAAGGAGGAA
m424:	(166)	TTATTATTAAGAGTCAAGGGGGTGTATGTTAGTGTATCGATTAAGTAAGGAGGAA
m427:	(166)	TTACTATTAAAAATTAACGAGGTATATACTAATGCATCGATCAAATAAGGAGGAG
m428:	(166)	TTATTATTAGAGACTAGAGAGGTATACACTAATGTACCGATTAAATAAGGGGGGA
m430:	(166)	TTATTATCAAAGACTAAAGAGGTATACATTAACGTATCGACTAAATGAGGAGGAA
m434:	(166)	TTATTACTAAAAGTTAAGGAGGTATATATTAATGTATCGATTAAATAAGGAGGAA
m435:	(166)	TTACTATCCAAAATCGAAGAGATACATACTAATGCATCGACTAAATAAGGGGGGA
m441:	(166)	CTATTATTGAAAACTGAAGAGGTATATACTGATGTACCGACTGAATAGGGAGGGA
m442:	(166)	TTATTATTAAAGACTGAAGAGGTGTATATTAATGTATCGATTAAATAAGGAGGAA
m444:	(166)	TCATCATTAAAAATTAAAGAGGCGCATATTAATGTATCGATTAAGTAAGGGGGAA
m445:	(166)	TTACTATTGAAAATTAAAGAGGTGTACATTGGTGCATCGATTAGATGAGGAGGAG
m447:	(166)	TTATTATTAGGGGTTAAAGAGGTACATATTAACGTATCGGTTAAATAAGGAGGGA
m449:	(166)	TTATTACTAAAAACTAGAGAGGTATATATTAATGTATCGATTAAATAAGGAGGAA
m454:	(166)	TTACTATTAAGAATTAAAGGGGTATATATTAATGTATCGACTAAATAAGGAGGAG
m459:	(166)	TTATCATTAAAAATTGAGGGGGTATGTACTAATGTGTCGGCTAAATAAGGAGGGA
m460:	(166)	TTATCATTAAGAATTGAAGAGGTGTATACTCGTGTGTCGATTAGATGAGGAGGAG
m463:	(166)	TTATTACTGAAGATTAGAGAGGTATATATTAATGTACCGGTTAAACAAGGAGGAA
m473:	(166)	TTATTGTTAAGAACTAAAGAGGTGTGTATTAATGTATCGACTAAGTAAGGAGGAA
m477:	(166)	TTGTTGTTAAAAATTAAAGAGGTATACATTAATGTATCGATTAAATGAGGAGGAA
m479:	(166)	TTATTGTTAAGAACTAAAGAGGTGTGTATTAATGTATCGACTAAGTAAGGAGGAA
m483:	(166)	TTATTATCAAAAGCTAAAGAGGTATATATTAATGCATCGATTAAGTAAGGGGGAA
m484:	(166)	TTACTATTAAAAGTTAAAGAGGTATGTATCAATGTATCGATTAAATAAGGAGGAA
m489:	(166)	TCGCTATTAAGAATTAAAGAGGTGTACACTAATGCATCGATTGGATAAGGAGGAA

Sequence alignment

```
m491: (166) TTATTATTAAGAGCTGAAGGGGTATGTATCAATGTATCGGTTAAGCAGGGAGGAG
m501: (166) TTATTATTAGAAGCTGGAGAGGTGCGTATCAACGTATCGACTAAATAAGGAGGAA
m505: (166) CTATTATTGAAAACCAGAGAGGTATGTACTAATGTATCGACCAAGTAAGGAGGAG
m509: (166) TTATTACCAAAAGTCAAAGAGGTGTGCACTGGCGTATCGGTTAGACAAGGAGGAG
m510: (166) TTATCACTAAGAATCAAAGAGGTATACACTAACGTATCGATTAAGCAAGGAGGAG
m514: (166) TTACTATCAAAAACTAGAGAGGTATATATTGGTGCATCGATTAAGTAAGGAGGAA
m517: (166) TTATTGTTAAAAATTAAAGGGGCATATGCTAATGTACCGATTAAGTAAGGAGGAA
m520: (166) TTACCATTAAGAATTAAAGAGGTATATGTCAATGTATCGATCAAGTAAGGAGGAA
m521: (166) TTATTATTAAAAATTGAAGAGGTATACACCAATGTACCGGTTGAATAGGGAGGGA
m524: (166) TTGTTACTGAGAATTAAAGAGGTATACACTAATGTACCGACTAAGTAAGGGGGAA
m526: (166) CTGCTACTAAAGATTAAAGAGGTATATATTAATGTATCGATTAAGTAAGGAGGAA
m534: (166) TCATTACCAAAAGTTAAAGAGGCATATATTAATGTATCGATTAAATAAGGAGGAA
m542: (166) TCATTATTAAGAATTAGAGAGATATCTATCGATGTATTGGTTAAATAAGGAGGAG
m546: (166) TTATTATTGAAAATTAAAGAGGTACATGCTACTGTATCAATTAAATAAGGAGGAG
m545: (166) TTATTGTTAGAGATTAAAGAGGTATGTGGTAATGTATCGATTGAATAGGGAGGAG
m548: (166) TTACTACCAAAAATCAAAGAGGTCTACATTGATGCATCGATTAAATAAGGAGGAA
m552: (166) TTGTTATTAAAAGCTAAAGAGGCATATATTAGTGCACCGACTAAATAAGGAGGGA
m565: (166) TTATTACCAAGAACTAAAGAGGTACATATTAATGCATCGATTAGGTAAGGAGGAA
m566: (166) CTGTCATTAAAAATCAAAACGGTATATACTAATGTATCGACTAAGTGAGGAGGAA
m573: (166) TTATCATTAAAGGTTAAAGAGGTAGATATTAATGTACCGATTAAATAAAGAGGAA
m580: (166) TTATGGTCAAGAATTAAGGAGGTATATATCAATGTATCGATCAAACAAGGAGGAG
m585: (166) TTATTGTTAAAAATCAAAGGGGCATCTATTGATGTATCGATTAAACAAGGGGGAA
m586: (166) TCACTATTAGAAATCAAAGAGGTGCACATTAGTGTACCGACTAAATAGGGAGGGA
m587: (166) TGATTATTAAGAGTTAAAGGGGTATATATTAATGTATCGATTAAATAAGGAGGGA
m590: (166) TTATTACTAAAGATTAAAGAGGTATATATTAACGCGTCGGCTAGACGAGGGGGAA
m591: (166) TCATTATTAGAAACTAGAGAGGTATATACTGATGTATCAATTAGATGAGGAGGAA
m599: (166) TTGTTATTAAAAATTAAAGAGGTATGTATTGGTGCATTGGTTGAATAAGGAGGAA
m606: (166) TTACCATTAAAAATTAAAGAGGTACATATTGATGTATCGATTAAATAAGGAGGGA
m626: (166) TTATTATTAACAATTAAAGAGGTATATATTAATGTATCGATTAAATAAGGAGGAA
m629: (166) TTATGATTCAAAATTAAAGAGGTATATATTAAGGTATCGATTAAATAAGGAGGAA
m640: (166) TTATGATTAAAAATTAAAGAGGTATATATTAATGTATCGATTAAATAAGGAGGAA
m647: (166) TTATTATTAAAAATTAAAGAGGTATATATTAATGTAGCGATTAACTAAGGAGGCA
m659: (166) TTATTATTCAAAATTAACGAGGTATATATTAATGTATCGATTAAATACGGAGGAA
m664: (166) TTATTATTAAAAATTAAAGAGGTATATATTAAGGTATCGATTAAATAAGGAGGAA
m670: (166) TTATTATTAAAAATTAAAGAGGTATATATTAATGTATCGATTAAATAAGGAGGAA
m675: (166) TTATTATTAAAAATTAAAGAGGTATATATTAATGTATCGATTCAATAAGGAGGAA
m701: (166) TTATTATTAAAAATTAAAGAGGTATATATTAATGTATCGATTAAATAAGGAGGAA
m702: (166) TTATTATTAAAAATTACAGAGGTATATATGAATGTATCGATTAAATCAGGAGGAA
m705: (166) TTATTATTAACAATTAAAGAGGTATATATTAATGTATCGATTAAATAAGGAGGAA
m706: (166) TTATTATTAAAAATTAAAGAGGTATATATTAATGTATCGATTAGATAAGGAGGAA
m708: (166) TTATCGTTAAAGATTAAAGAGGCACATATTAATGTGTCGATTAAATGAGGAGGAG
m709: (166) GTATTCTTACAAATTAAAGAGGTATCTATTAATGTATCGAGTACATAAGGAGGAA
m710: (166) TTATTATTAAAAATTAAAGAGGTATATATTAATGTATCGATTAAATAAGGAGGAA
```

	(221)	221 224
m000:	(221)	TAAA
m001:	(221)	TAAA
m003:	(221)	TAGC
m004:	(221)	CAGA
m005:	(221)	TAGA
m006:	(221)	TAAA
m007:	(221)	TAAA
m010:	(221)	TAAA
m014:	(221)	CAAA
m015b:	(221)	TCAA
m017:	(221)	TAGA
m018:	(221)	TAAA
m019:	(221)	TAAA
m021:	(221)	TAAG
m024:	(221)	TGGG
m026:	(221)	TCAG
m028:	(221)	TAAA
m029:	(221)	TAAA
m030:	(221)	TAAA
m031:	(221)	TGAG
m054:	(221)	TGAA
m085:	(221)	TAAG
m092:	(221)	TAAA
m150:	(221)	CAAA
m198:	(221)	TAAG
m213:	(221)	TAAA
m232:	(221)	TAAA
m244:	(221)	TAAA
m354:	(221)	TAGA
m360:	(221)	TAAA
m363:	(221)	TAAA
m396:	(221)	TAAA
m412:	(221)	TAAG
m413:	(221)	TAGA
m421:	(221)	TAGA
m424:	(221)	TAAA
m427:	(221)	TAAA
m428:	(221)	CAAA
m430:	(221)	TAAG
m434:	(221)	TAGA
m435:	(221)	TAAA
m441:	(221)	TAGA
m442:	(221)	TAAA
m444:	(221)	TAAA
m445:	(221)	TAGG
m447:	(221)	TAGA
m449:	(221)	TGAA
m454:	(221)	TAAG
m459:	(221)	TGAG
m460:	(221)	TAAG
m463:	(221)	TAAA
m473:	(221)	TAAA
m477:	(221)	TAAA
m479:	(221)	TAAA
m483:	(221)	TAAA
m484:	(221)	TGAA
m489:	(221)	TGGA

m491: (221) TAAG
m501: (221) TAAA
m505: (221) TAAG
m509: (221) TAAA
m510: (221) TAAA
m514: (221) TAAA
m517: (221) TGGA
m520: (221) TAAA
m521: (221) TAAA
m524: (221) TAAA
m526: (221) TAAA
m534: (221) TGAA
m542: (221) CAGA
m546: (221) TGAA
m545: (221) TGAA
m548: (221) TAAA
m552: (221) TAAG
m565: (221) TAAA
m566: (221) TAGA
m573: (221) CAAA
m580: (221) TAAG
m585: (221) TAGA
m586: (221) TAAA
m587: (221) TAAA
m590: (221) CAAA
m591: (221) TAAA
m599: (221) TAAG
m606: (221) TAAA
m626: (221) TAAA
m629: (221) TAAA
m640: (221) TAAA
m647: (221) TAAA
m659: (221) TAAA
m664: (221) TAAA
m670: (221) TAAA
m675: (221) TAAA
m701: (221) TAAA
m702: (221) TAAA
m705: (221) TAAA
m706: (221) TAAA
m708: (221) TAGA
m709: (221) TAAA
m710: (221) TAAA
