# Supplementary material for: Quantitative Design of Regulatory Elements Based on High-Precision Strength Prediction Using Artificial Neural Network
Source: PLoS One. 2013 Apr 1;8(4):e60288. doi: 10.1371/journal.pone.0060288 (PMC3613377; doi:10.1371/journal.pone.0060288)
Supplement: Dataset S1 — Training data set and test data set for model NET90_19_576. (DOCX) [file pone.0060288.s004.docx]

**Dataset S1. Training data set and test data set for model NET90_19_576.**

**Training data set:**

m000, m001, m003, m004, m006, m007, m010, m014, m015b, m018, m019, m021, m024, m026, m028, m029, m030, m054, m085, m092, m150, m198, m213, m232, m244, m354, m360, m363, m396, m412, m413, m421, m424, m427, m428, m434, m435, m441, m442, m444, m445, m447, m449, m454, m460, m463, m473, m477, m479, m483, m484, m489, m491, m501, m509, m510, m514, m517, m520, m521, m534, m542, m545, m546, m548, m552, m565, m566, m573, m580, m585, m586, m587, m590, m599, m606, m626, m629, m640, m647, m659, m664, m670, m701, m702, m705, m706, m708, m709, m710

**Test data set:**

m005, m017, m031, m430, m459, m505, m524, m526, m591, m675
